# Global trends in patient-reported outcomes and quality-of-life research for breast cancer: a 20-year bibliometric study

**DOI:** 10.1007/s10552-025-02097-x

**Published:** 2026-01-17

**Authors:** Meng Li, Xiongxiong Li, Jin Shang

**Affiliations:** Breast Disease Center, Xi’an People’s Hospital (Xi’an Fourth Hospital), Xi’an, 710004 China

**Keywords:** Breast cancer, Patient-reported outcome, Quality of life, Bibliometric analysis

## Abstract

**Objective:**

To conduct a 20-year bibliometric analysis of patient-reported outcomes (PROs) and quality-of-life (QoL) research in breast cancer, with focus on evolving priorities in women-centered care, global equity gaps, and implications for survivorship policy.

**Methods:**

A bibliometric analysis of publications from 2005 to 2024 was conducted using Web of Science. Following clear inclusion criteria (English articles focusing on PROs/QoL in breast cancer), we analyzed 1857 publications using CiteSpace and VOSviewer to map co-citation networks, keyword bursts, and collaboration patterns.

**Results:**

A bibliometric analysis of 1,857 publications (2005–2024) revealed exponential growth in PROs and QoL research for breast cancer, with a notable surge after 2012. The United States led in output (30%) and influence, while China ranked second in volume but demonstrated lower citation impact. European nations such as France and Spain exhibited high per‑paper influence. Memorial Sloan Kettering Cancer Center was the most productive institution, yet the University of California, San Francisco achieved the highest average citations per paper. Supportive Care in Cancer published the most studies, while the Journal of Clinical Oncology was the most co‑cited. Seminal works—including the BREAST‑Q and EORTC QLQ modules—were the most frequently co‑cited references. Keyword evolution reflected a conceptual shift: early research emphasized “randomized trial” and “adjuvant therapy,” whereas recent trends prioritize “mental health” and “immediate breast reconstruction,” signaling a broader integration of psychosocial and patient‑centered outcomes.

**Conclusions:**

While innovations in PRO tools and survivorship science mark clear progress, the field remains constrained by a Western-centric paradigm that perpetuates inequities in addressing the needs of racial minorities, young patients with fertility concerns, and populations in resource-limited settings. Future efforts require inclusive research governance with strengthened LMIC leadership, contextualized priority-setting to bridge cross-cultural validity gaps, and strategic digital health integration to advance equitable patient-centered care beyond survival metrics.

**Supplementary Information:**

The online version contains supplementary material available at 10.1007/s10552-025-02097-x.

## Introduction

Breast cancer causes nearly one in four cancer cases and one in six cancer deaths in women worldwide. In 2022, it impacted 2.3 million women globally [1]. Due to major advances in surgery, the 5-year survival rate in high-income countries is now 90% [2]. However, now, extended survival has changed the clinical goals. The focus is no longer just on living longer; it is about improving the quality of life (QoL) [3]. Patient-reported outcomes (PROs) are health data provided directly by patients, without clinician input [4]. They are important for measuring surgical success. They also help in deciding on breast-conserving surgery, reconstruction, and survivorship care [5].

Despite the consensus on the utility of PROs, critical gaps persist. Different PRO instruments, such as EORTC QLQ-BR23 and FACT-B, make it tough to compare studies [6, 7]. This is especially true in multicultural groups. Early studies mainly looked at physical outcomes such as pain and lymphedema. New evidence shows that psychosocial factors, such as body image and sexual health [8], also matter. This shift calls for longer-term research frameworks [9]. Third, technological changes—like telemedicine and AI-driven PROs platforms [10, 11]—are changing research methods. However, their bibliometric impacts are still unclear.

The current literature covers patient-reported outcomes (PROs) in oncology [12]. However, no studies have tracked the development of PRO research focused on breast surgery. There are many ways to map an academic field. Recently, bibliometric analysis has become the leading method [13]. This shift is thanks to advances in math and computer techniques. Bibliometrics uses numbers to analyze publication patterns. This helps us understand trends, collaborations, and knowledge structures [14].

This study examines trends in publication volume, geography, and interdisciplinary links over the past two decades. It will find research hotspots and changes over time using keyword co-occurrence and citation bursts. Next, it will give tips on how to better use patient-reported outcomes (PROs) in breast cancer research. This aims to strengthen global collaboration.

## Materials and methods

### Data collection

The Web of Science Core Collection (WoSCC) database is more complete and accurate than others. This makes it the top choice for systematic literature analysis [15]. Thus, this study selected WoSCC as the primary data source for bibliometric analysis.

Search Strategy: Publications were retrieved from the Web of Science core collection using a structured Boolean query. The search was executed on 5 March, 2025. The search targeted records containing terms for (1) patient-reported outcomes (e.g., PROMs), (2) quality of life (e.g., well-being), and (3) breast cancer (e.g., mastectomy). Specific exclusion terms were applied to refine the results. The complete search strategy is available in the Supplementary Appendix.

**Fig. 1:**
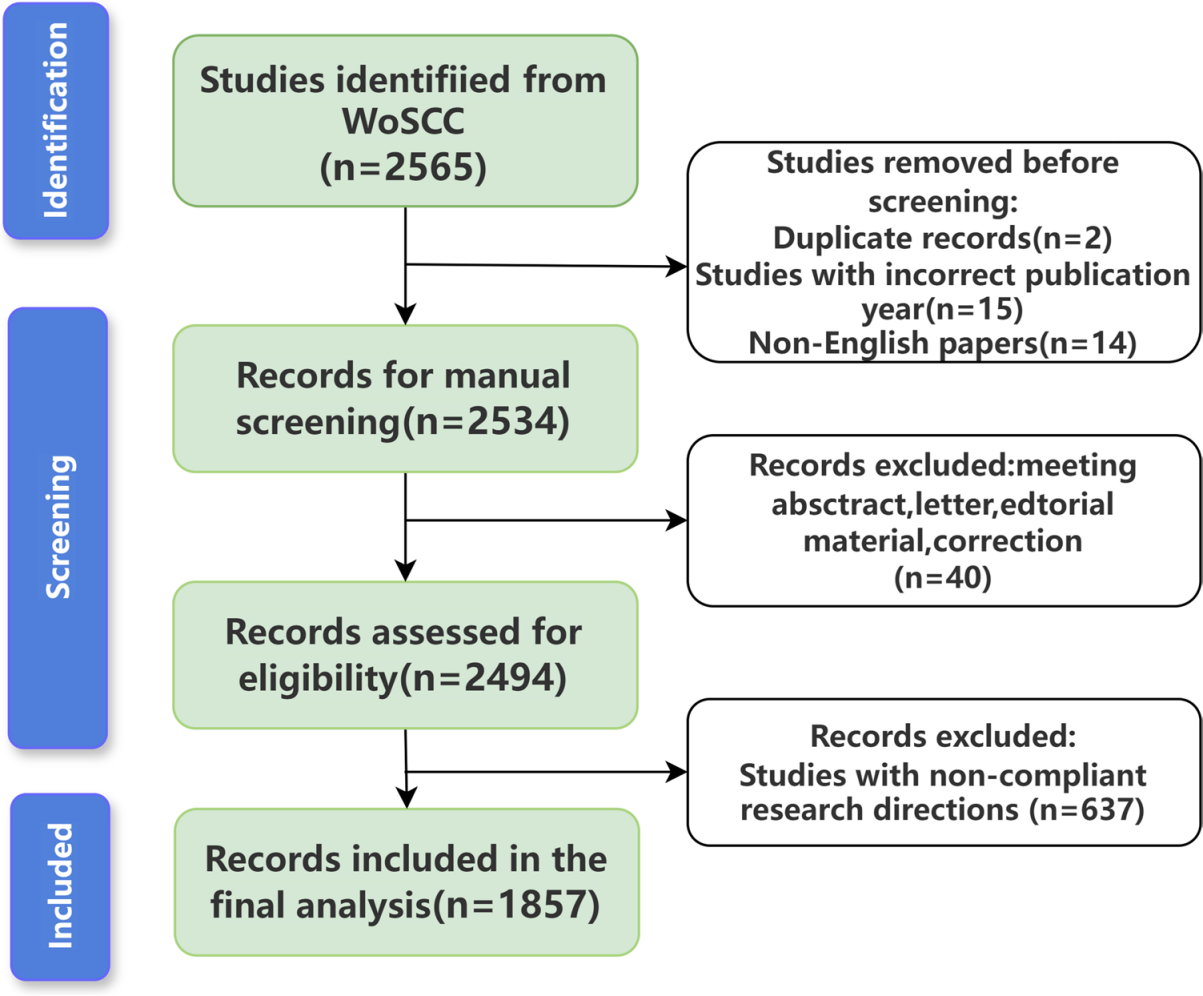
Flowchart of literature selection

Researchers select studies based on the following criteria: (1) full-text, peer-reviewed journal articles; (2) focusing on PROs or QoL in breast cancer; (3) articles in English; (4) publications must be from 1 January, 2005, to 31 December, 2024. Exclusion criteria included: (1) studies not focused on patient-reported outcomes (PROs) or quality of life (QoL) for breast cancer patients; (2) duplicate publications; (3) document types such as meeting abstracts, editorials, letters, and commentaries. Figure 1 illustrates the specific flow.

Data from the included literatures underwent a thorough extraction and analysis process. Data extracted included: country of origin, institution, author names, journal title, cited references, and keywords. Bibliographic records from WoSCC were downloaded in plain text format, including full records and citations. The screening process was conducted by one reviewer and independently verified by a second reviewer. A consistency check was performed on a random sample of records to confirm reliability.

### Bibliometric analysis

All valid data from WoSCC went into CiteSpace (6.4.R1 (64-bit) Advanced) and VOSviewer (1.6.20). This helped create visual graphs for both quantitative and qualitative analysis.

CiteSpace (6.4.R1) is a Java tool created by Professor Chaomei Chen at Drexel University. It uses co-citation analysis, burst detection, and time-slicing algorithms. These features help map how knowledge structures change and identify new research areas [16]. This software tracks citation trends over time. It finds key studies, important shifts, and new technologies. This helps researchers foresee major scientific changes and innovation trends.

VOSviewer (1.6.20) is a free, Java-based tool for bibliometric visualization. Developed in 2009 by Ludo Waltman and Nees Jan van Eck at Leiden University, it helps users create and analyze large bibliographic networks [17]. The tool uses clustering and scaling techniques to create interactive maps that display research collaboration, citation links, and keyword patterns in literature data.

CiteSpace (6.4.R1) helped analyze the literature. It created maps of countries and institutions, authors, and journals. It also created co-citation clusters and keyword citation bursts. VOSviewer (1.6.20) visualized the co-occurrence of institutions, authors, journals, and references. Each point on the graph represents a country, institution, author, or journal. These points cluster into groups based on collaboration. Point size indicates article output; connection strength reflects collaboration frequency.

Additionally, R-Bibliometrix and R version 4.4.3 were utilized for data processing and chord diagram visualization.

## Results

### General data and annual output

The bibliometric analysis included 1,857 publications from 2005 to 2024. It covered 74 countries, 3,134 institutions, 10,689 authors, and 48,893 cited references.

**Fig. 2:**
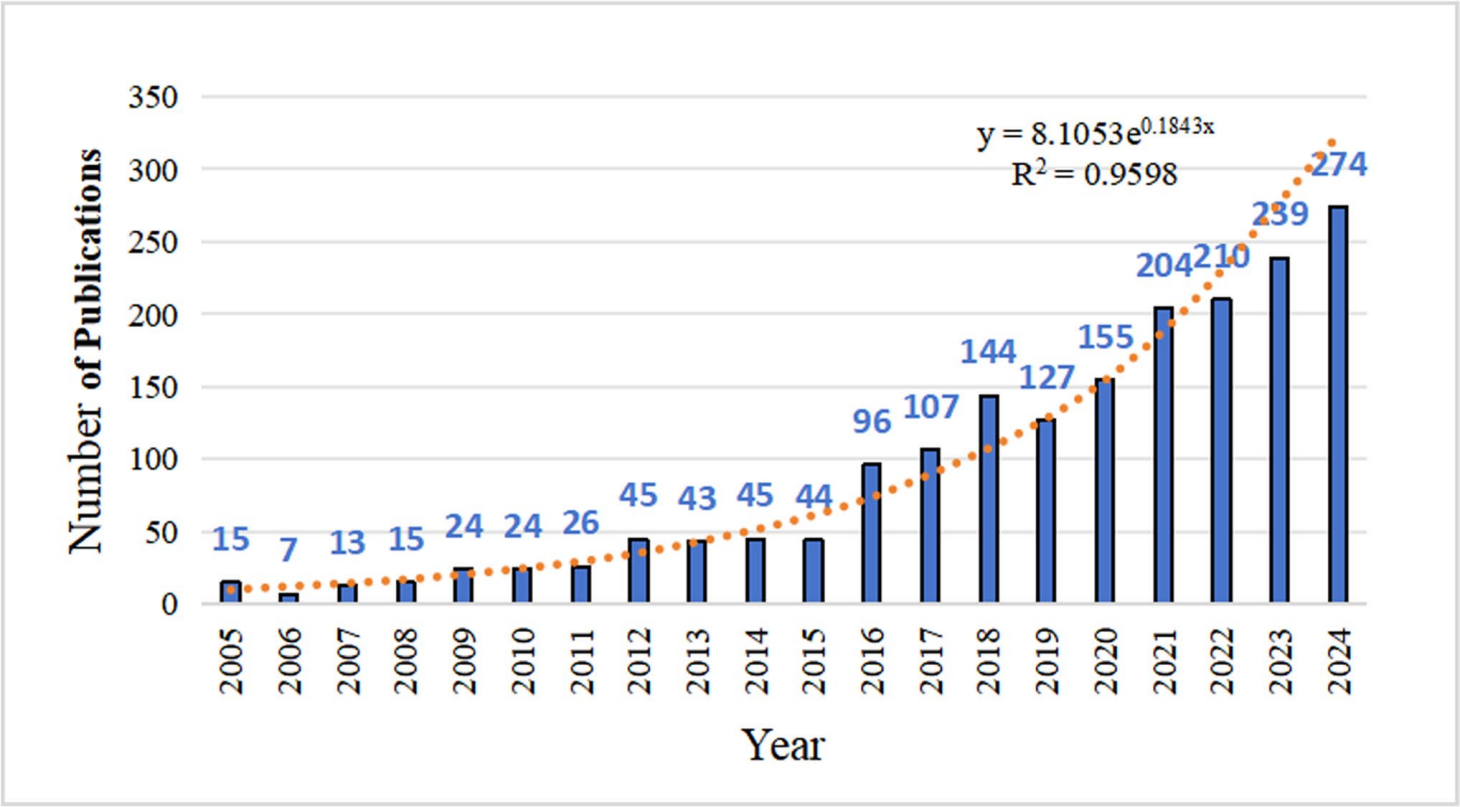
Annual number of publications

The number of annual publications on quality of life (QoL) and patient-reported outcomes (PROs) for breast cancer patients increased significantly from 2005 to 2024 (Fig. 2). From 2005 to 2011, the number of publications stayed low, ranging from 7 to 26 per year. Then, after 2012, it began to rise steadily. A notable surge occurred in 2016 (n = 96), with peak activity observed in recent years. The exponential growth aligns with broader trends in oncology, emphasizing patient-centered outcomes.

### Analysis of countries

**Fig. 3:**
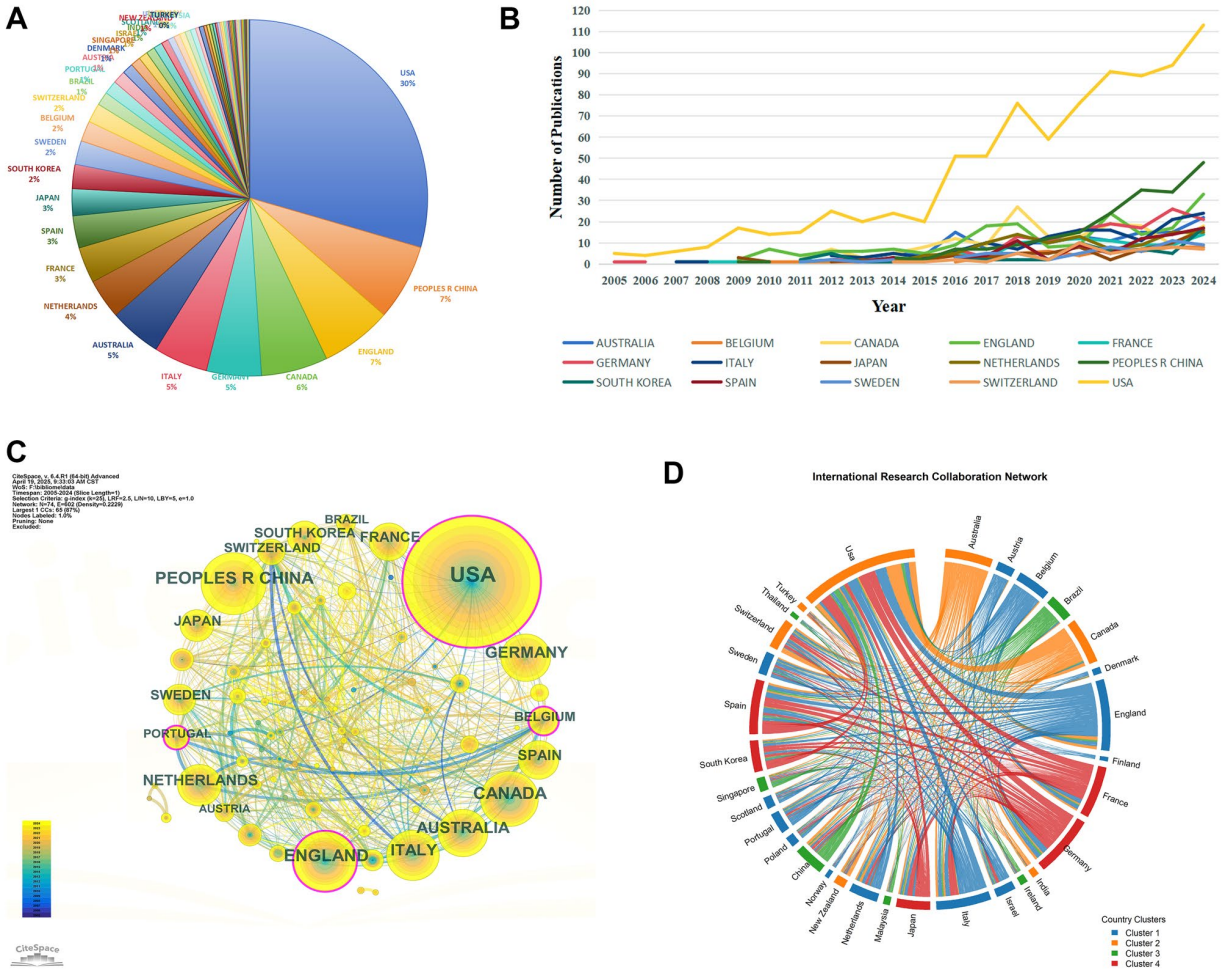
Analysis of countries. **A** The pie chart showing the percentage of published literature from each country. **B**. Line graph of national publications. **C** Networks of country cooperation. D Chord Diagram of country collaboration networks

The pie chart (Fig. 3A) reveals distinct global contributions and research impact in the field. The United States, contributing 30% of total publications, not only leads in output but also demonstrates robust research influence with an average of 33.18 citations per paper (Table 1). China, responsible for 7% of the output, shows significant quantitative production, though its average citation rate (12.56) suggests a potential gap in immediate scholarly impact. England (7%) and Canada (6%) further solidify their key roles, with Canada, in particular, achieving a notable average of 43.94 citations per paper.

**Table 1:** Top 10 countries

Rank	Country	Number of Documents	Total Citations	Average Citations	Total Link Strength
1	USA	845	28,033	33.18	648
2	Peoples R China	196	2461	12.56	164
3	England	185	5213	28.18	388
4	Canada	176	7734	43.94	251
5	Germany	142	6488	45.69	335
6	Italy	142	4014	28.27	299
7	Australia	137	3909	28.53	259
8	Netherlands	104	2525	24.28	158
9	France	89	4684	52.63	283
10	Spain	83	4137	49.84	299

European nations present a varied profile. Germany and Italy each contributed 5% of publications, with Germany achieving a high average of 45.69 citations. Similarly, Australia (5%) maintained a solid average of 28.53 citations. The collective output (15%) from the Netherlands (4%, avg. 24.28 citations), France (3%, avg. 52.63 citations), and Spain (3%, avg. 49.84 citations) is particularly noteworthy. France and Spain stand out with exceptional per-paper influence, each nearing 50 citations, rivaling the impact of leading countries despite their smaller volume.

Meanwhile, Asian engagement remains emerging but limited. Japan contributed 3% and South Korea 2% of the total output. Their research impact, as reflected in citation rates, appears less pronounced relative to the major contributing nations.

The longitudinal analysis (Fig. 3B) reveals three distinct patterns of research engagement: Sustained leadership: The USA showed steady growth during the study period, with particularly accelerated output after 2015. China exhibited exponential growth since 2015, surpassing several European nations by 2020. Stable contributors include Canada, Germany, and Australia. These countries showed steady annual growth. They usually produced 5–20 publications each year after 2010. Intermittent participants: Many European countries, such as Sweden and Switzerland, along with some Asian nations, showed varying output. They rarely had more than 10 publications each year.

The Collaboration Network Map (from CiteSpace) (Fig. 3C) shows Core Collaboration Groups: South Korea, France, and Switzerland have formed a unique triangular partnership, highlighting their strong cross-regional research synergy. The USA has a radial network that includes Germany, Belgium, Spain, Canada, Australia, and Italy, underscoring its global scientific hub role. China serves as a key hub, forming multilateral ties with Japan, Sweden, Portugal, the Netherlands, Austria, and England. Brazil and Switzerland work together in specific areas, which are noted separately. This map illustrates the structure of global research collaboration. It highlights the strong influence of core nations such as the USA and China. It also highlights partnerships between regions such as South Korea, France, and Switzerland. Figure 3D displays the VOSviewer Data-Driven Chord Diagram (R-generated). It illustrates research collaborations between 31 countries. The thickness of the edges shows how intense the collaborations are (Value). The United States (USA) is the central hub, with the strongest ties to Canada (Value = 90), England (60), and Germany (52). Key regional clusters include: Europe-centric: Germany, France, Switzerland; Anglosphere: USA, Canada, Australia, England; Asia–Pacific: China, Japan, South Korea. The two images provide complementary insights: the former highlights structural hierarchies, while the latter measures collaboration intensity.

### Analysis of institutions

While Table 2 confirms the leading role of U.S. institutions, which constitute eight of the top ten by publication volume, the incorporation of average citations per paper reveals a more nuanced landscape of research impact. Memorial Sloan Kettering Cancer Center (USA) ranked first in productivity (138 documents), yet the University of California, San Francisco (UCSF) demonstrated the highest scholarly influence with a markedly superior average of 74.44 citations per paper. Other institutions that combined strong output with high per-article impact include the University of Michigan (71 documents, 56.62 avg. citations) and Canada’s McMaster University (52 documents, 53.13 avg. citations). The presence of The University of Sydney (Australia) also highlights active non-U.S. contributions. This analysis underscores that normalized metrics are crucial for evaluating research influence beyond sheer output volume.

**Table 2:** Top 10 institutions

Rank	Institution	Country	Number of Documents	Total Citations	Average Citations	Total Link Strength
1	Memorial Sloan Kettering Cancer Center	USA	138	7028	50.91	307
2	The University of Texas MD Anderson Cancer Center	USA	79	3879	49.10	222
3	University of Michigan	USA	71	4020	56.62	174
4	Mayo Clinic	USA	56	1760	31.43	193
5	Harvard Medical School	USA	55	1138	20.69	178
6	McMaster University	Canada	52	2763	53.13	161
7	Dana-Farber Cancer Institute	USA	50	1887	37.74	269
8	University of Toronto	Canada	50	1947	38.94	146
9	University of California, San Francisco (UCSF)	USA	45	3350	74.44	187
10	The University of Sydney	Australia	44	1185	26.93	151

**Fig. 4:**
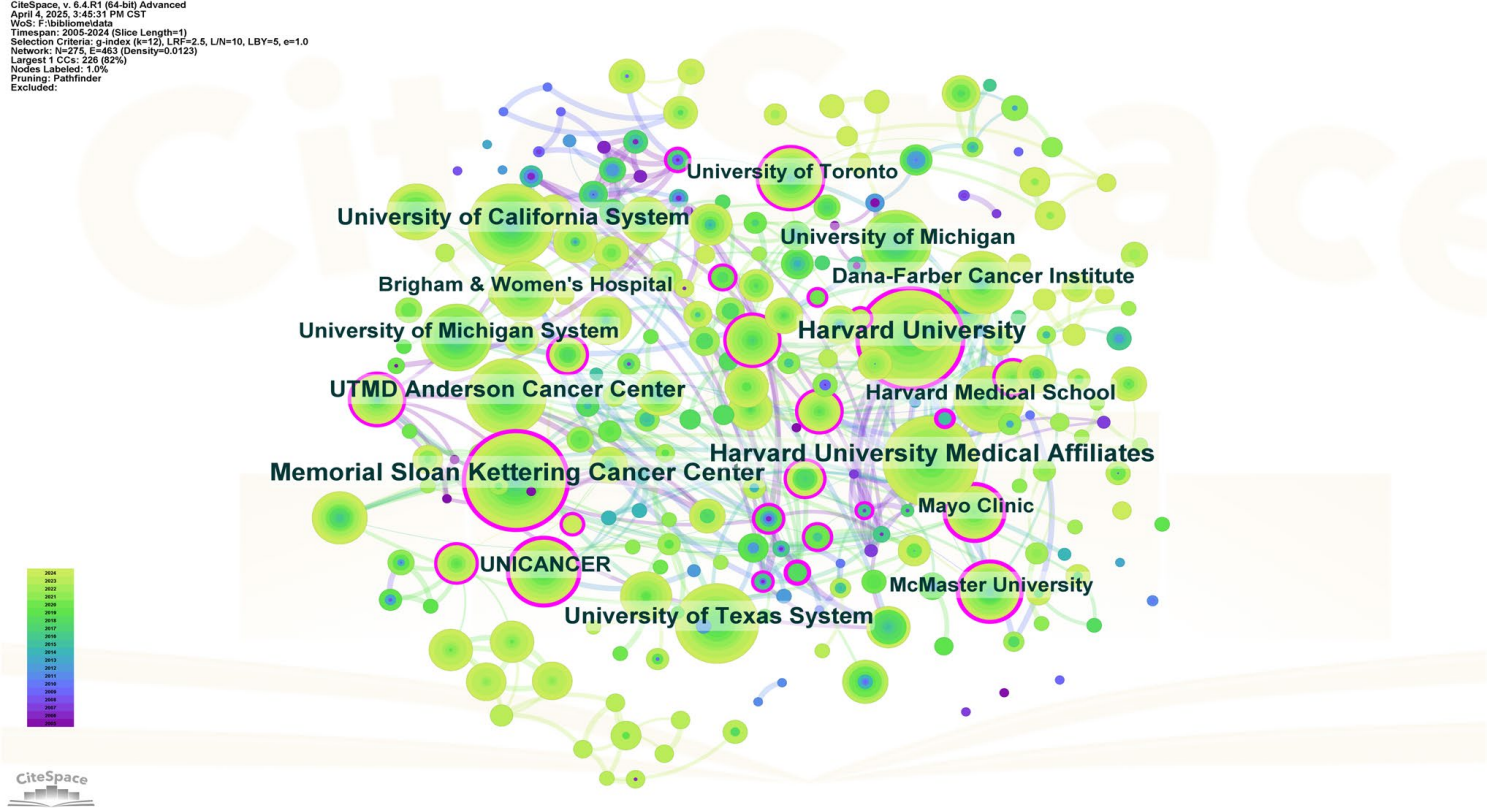
The co-occurrence network map of institutions

**Fig. 5:**
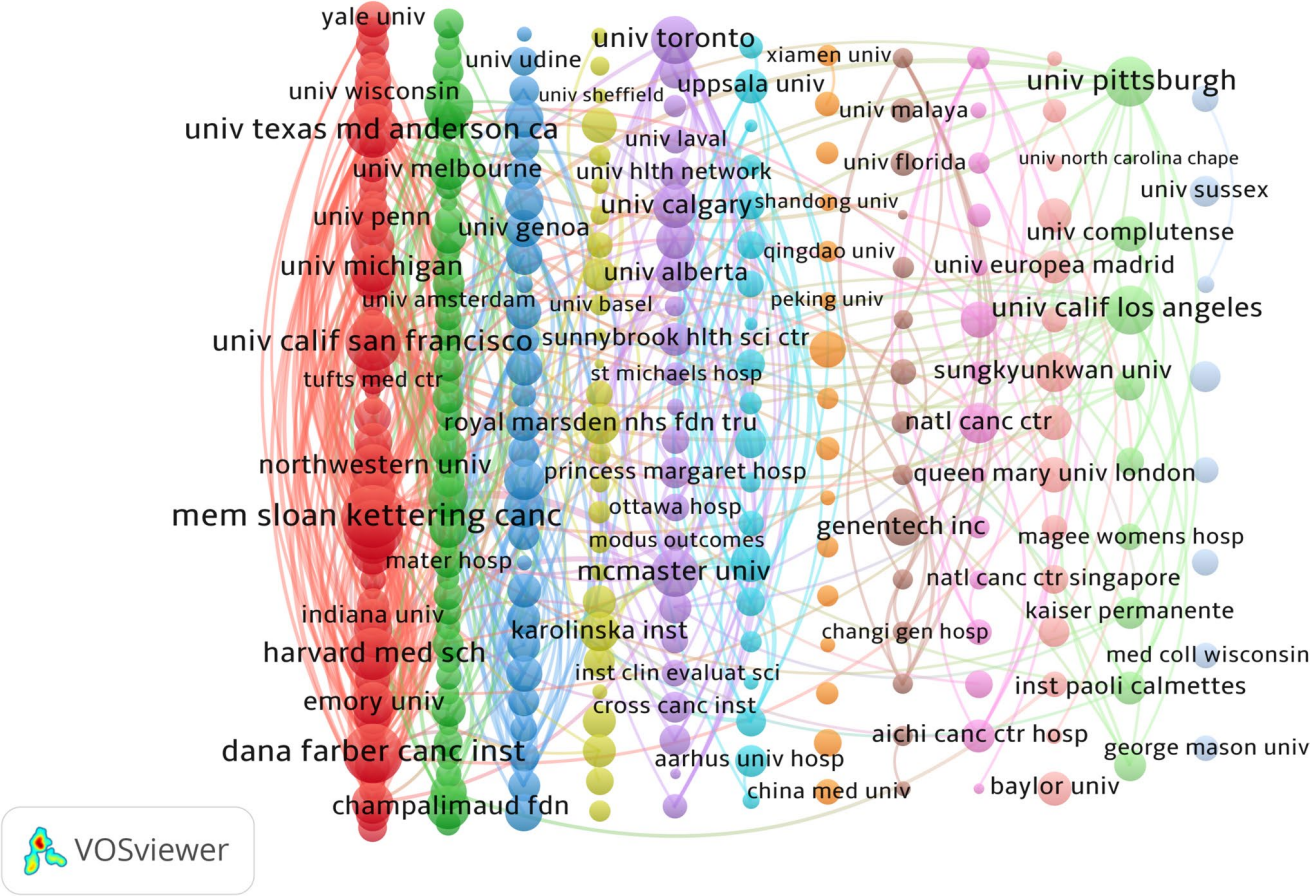
The collaboration network map of institutions

Figure 4 illustrates the co-occurrence mapping of cooperation among institutions. Dana-Farber Cancer Institute was the top node, with a centrality of 0.30. It greatly outperformed other institutions. This positions it as a global hub integrating diverse research clusters. Lower-centrality institutions, such as Harvard affiliates, UNICANCER, and the University of Sydney, had centrality scores of 0.07 to 0.09. They showed weaker inter-cluster connectivity. This suggests they focus more on activities within their own clusters. The analysis of institutions with over five articles created a network map showing collaborations among 295 institutions (Fig. 5). This map reveals that these collaborations are mainly grouped into 12 clusters.

### Analysis of journals and co-cited journals

**Fig. 6:**
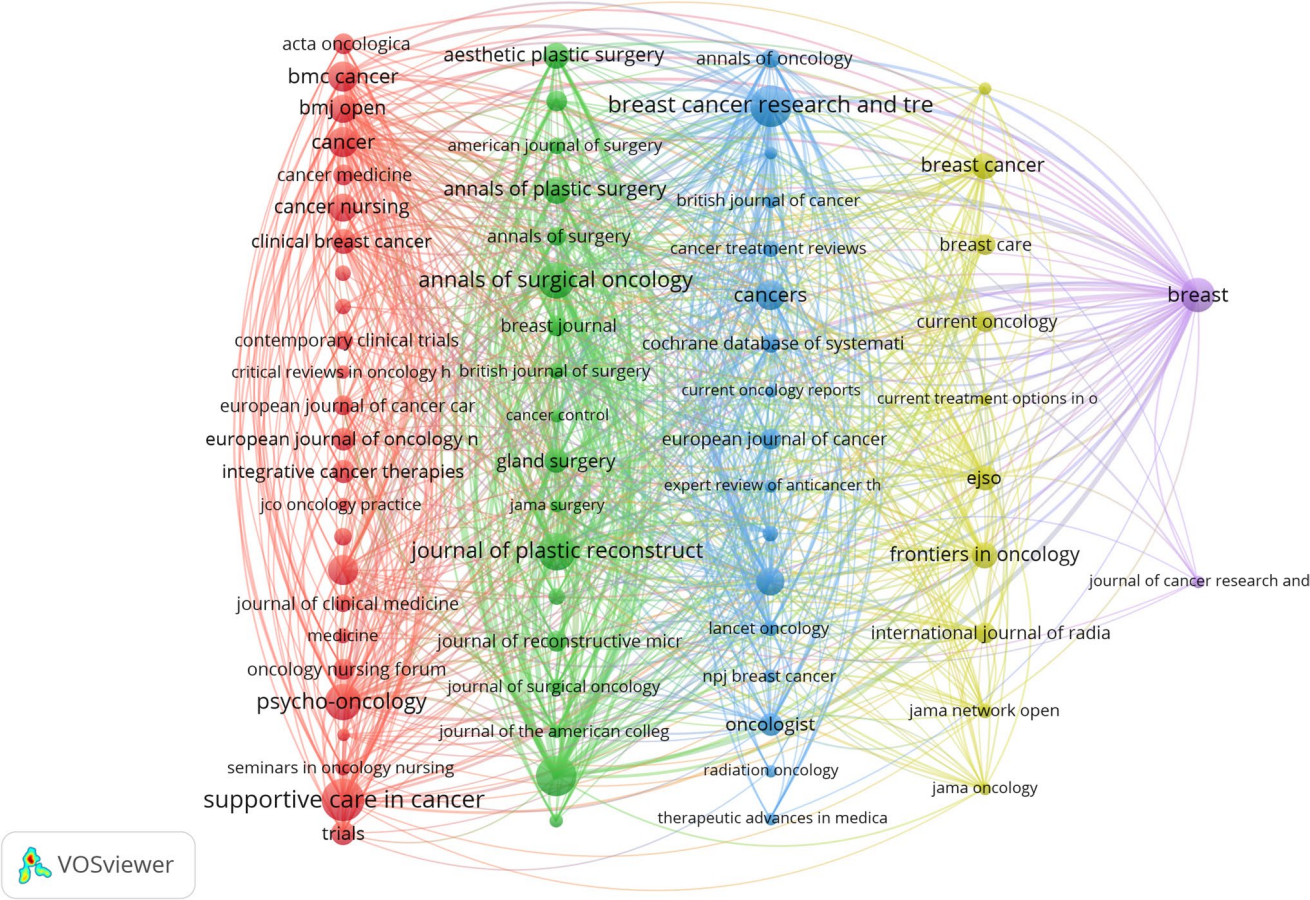
The network visualization diagram of journals

All the searched literatures came from 237 journals. Table 3 ranks journals by research output and displays key metrics including Impact Factor (IF), JCR ranking, and H-index. *Supportive Care in Cancer* leads with 99 publications, followed by *Breast Cancer Research and Treatment* (94) and *Plastic and Reconstructive Surgery* (90). The highest IF is held by *Cancer* (6.1), which also had the top H-index (277). Q1-ranked journals also include *Plastic and Reconstructive Surgery* (IF 3.2), *Breast* (IF 5.7), and *Cancers.* Most others fall under Q2 (e.g., *Supportive Care in Cancer,* IF 2.8). Higher H-index values indicate a lasting citation impact. This holds true even for journals with a moderate impact factor (IF). For instance, *Breast Cancer Research and Treatment* had an IF at 3.0 and an H-index of 139. More publications, such as *Supportive Care in Cancer* with 99, do not always indicate higher impact factors (IF) or better rankings. For example, *Cancer* had the lowest output (43) but the highest IF (6.1) and H-index (277), indicating strong influence. We filtered the journals with at least 5 publications yielded 72 journals, which were plotted using VOSviewer in Fig. 6**.**

**Table 3:** Top 10 Journals

Rank	Journal	Output	IF	JCR	H-index
1	Supportive Care in Cancer	99	2.8	Q2	98
2	Breast Cancer Research and Treatment	94	3.0	Q2	139
3	Plastic and Reconstructive Surgery	90	3.2	Q1	160
4	Journal of Plastic Reconstructive and Aesthetic Surgery	70	2.0	Q3	82
5	Annals of Surgical Oncology	69	3.4	Q2	155
6	Psycho-Oncology	68	3.3	Q2	123
7	Breast	56	5.7	Q1	70
8	Cancers	44	4.5	Q1	53
9	Journal of Cancer Survivorship	44	3.1	Q2	53
10	Cancer	43	6.1	Q1	277

**Fig. 7:**
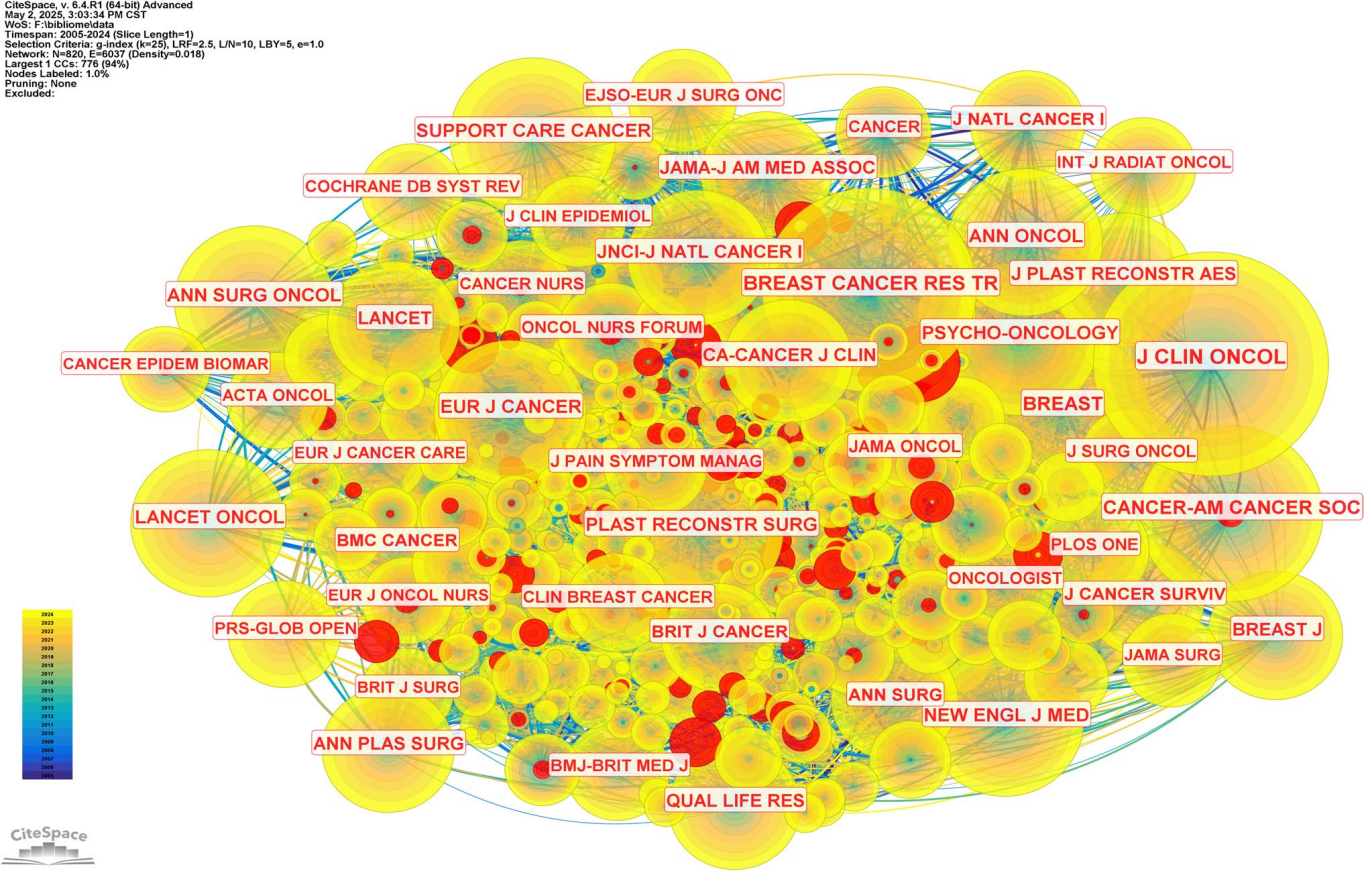
The co-citation network map of journals

Co-citation frequency serves as a key indicator of a journal’s scientific influence. Table 4 lists journals ranked by their co-citation frequency. The Journal of Clinical Oncology leads with 3356 citations. Next is Plastic and Reconstructive Surgery at 3,030 citations. Breast Cancer Research and Treatment follows with 2,646 citations. Annals of Oncology has the highest IF at 56.7. Meanwhile, the Journal of Clinical Oncology boasts the top H-index of 494. Most journals are classified as Q1 or Q2. Figure 7 visualizes journal co-citation networks, emphasizing the journals that are frequently co-cited and illustrating their relationships.

**Table 4:** Top 10 Co-cited Journals

Rank	Cited Journal	Citation	IF	JCR	H-index
1	Journal of Clinical Oncology	3356	42.1	Q1	494
2	Plastic and Reconstructive Surgery	3030	3.2	Q1	160
3	Breast Cancer Research and Treatment	2646	3.0	Q2	139
4	Psycho-Oncology	2287	3.3	Q2	123
5	Cancer	2053	6.1	Q1	277
6	Supportive Care in Cancer	2028	2.8	Q2	98
7	Annals of Surgical Oncology	1766	3.4	Q2	155
8	Journal of Plastic Reconstructive and Aesthetic Surgery	1650	2.0	Q3	82
9	Breast	1157	5.7	Q1	70
10	Annals of Oncology	1119	56.7	Q1	210

**Fig. 8:**
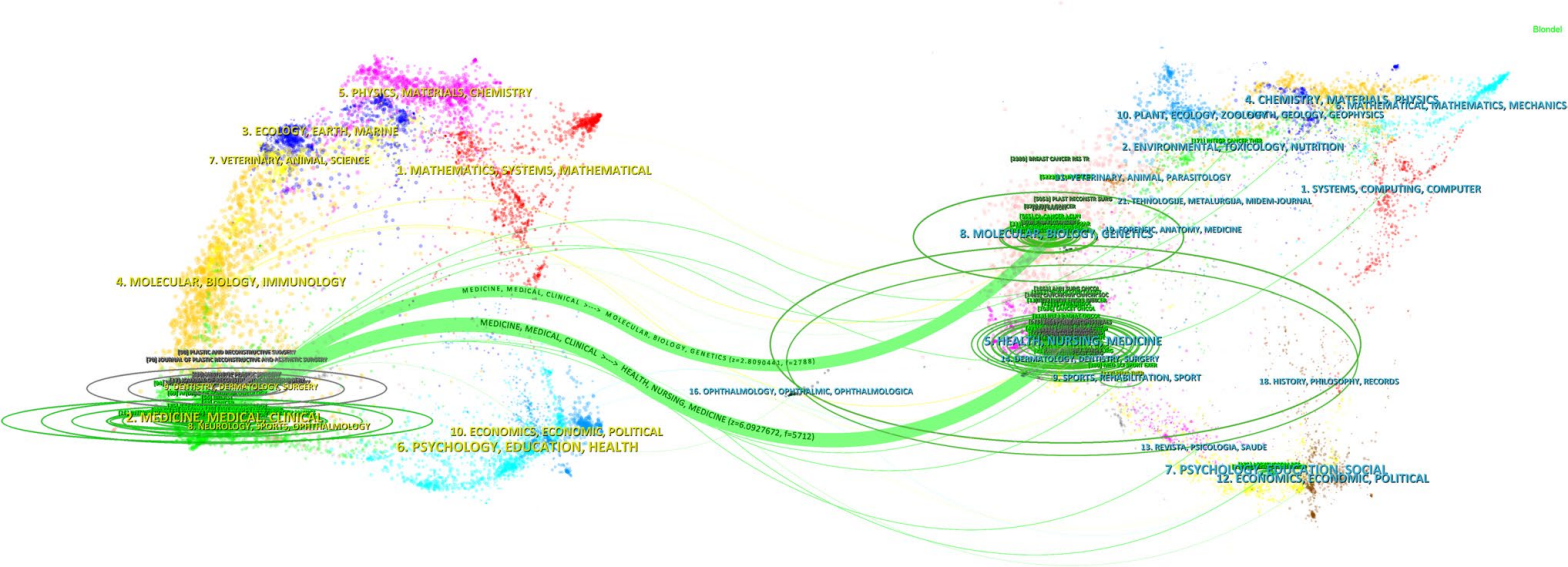
The dual-map overlay of journals

The thematic distribution of academic publications is illustrated through a dual-map overlay. The colored path indicates citation connections, with citing journals on the left and cited journals on the right. Figure 8 highlights a main citation pathway. Most papers focused on "molecular biology, immunology, and clinical medicine." The cited literature was mostly in "molecular biology, immunology," and "medicine, medical, clinical."

### Authors and co-cited authors

**Fig. 9:**
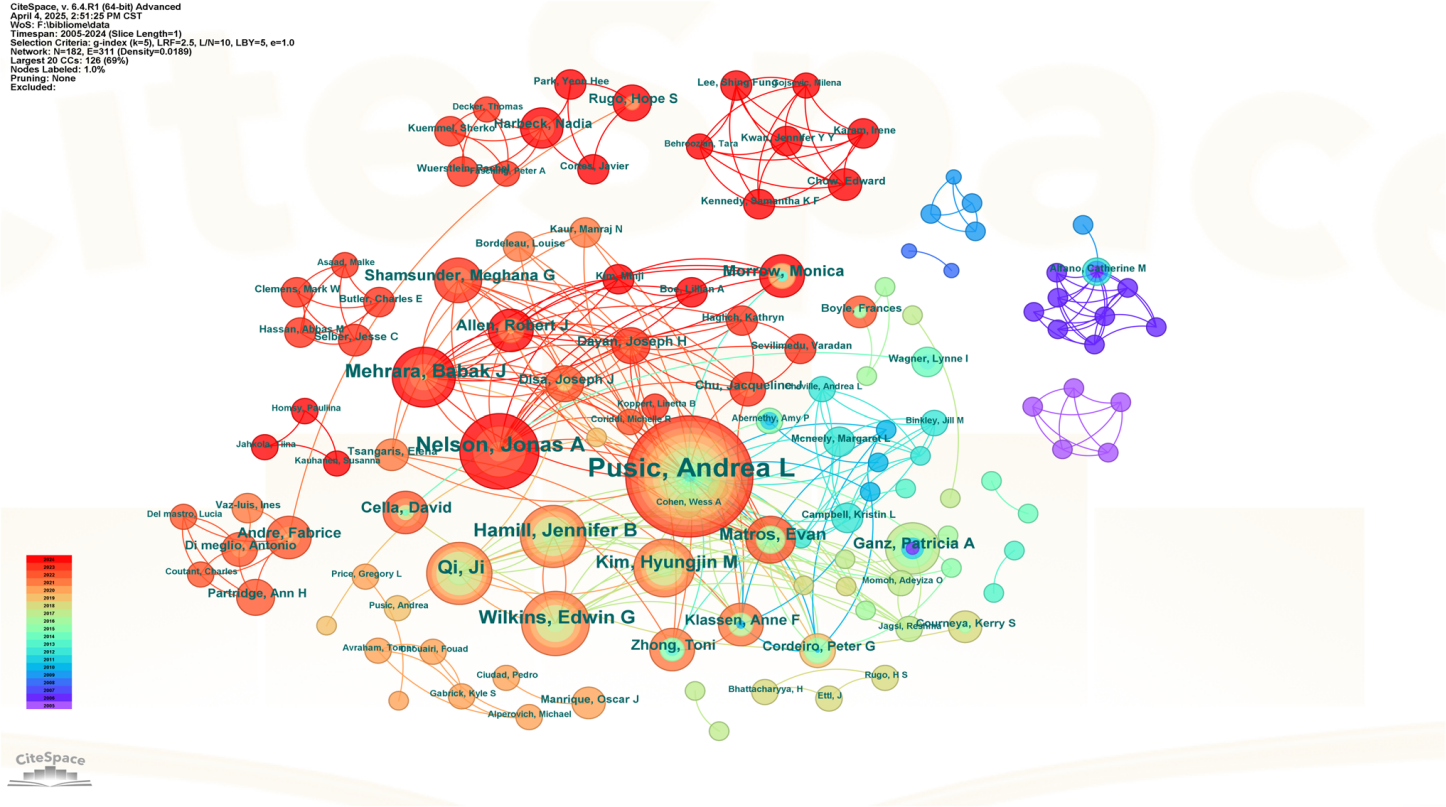
The cooperation network map of authors

**Fig. 10:**
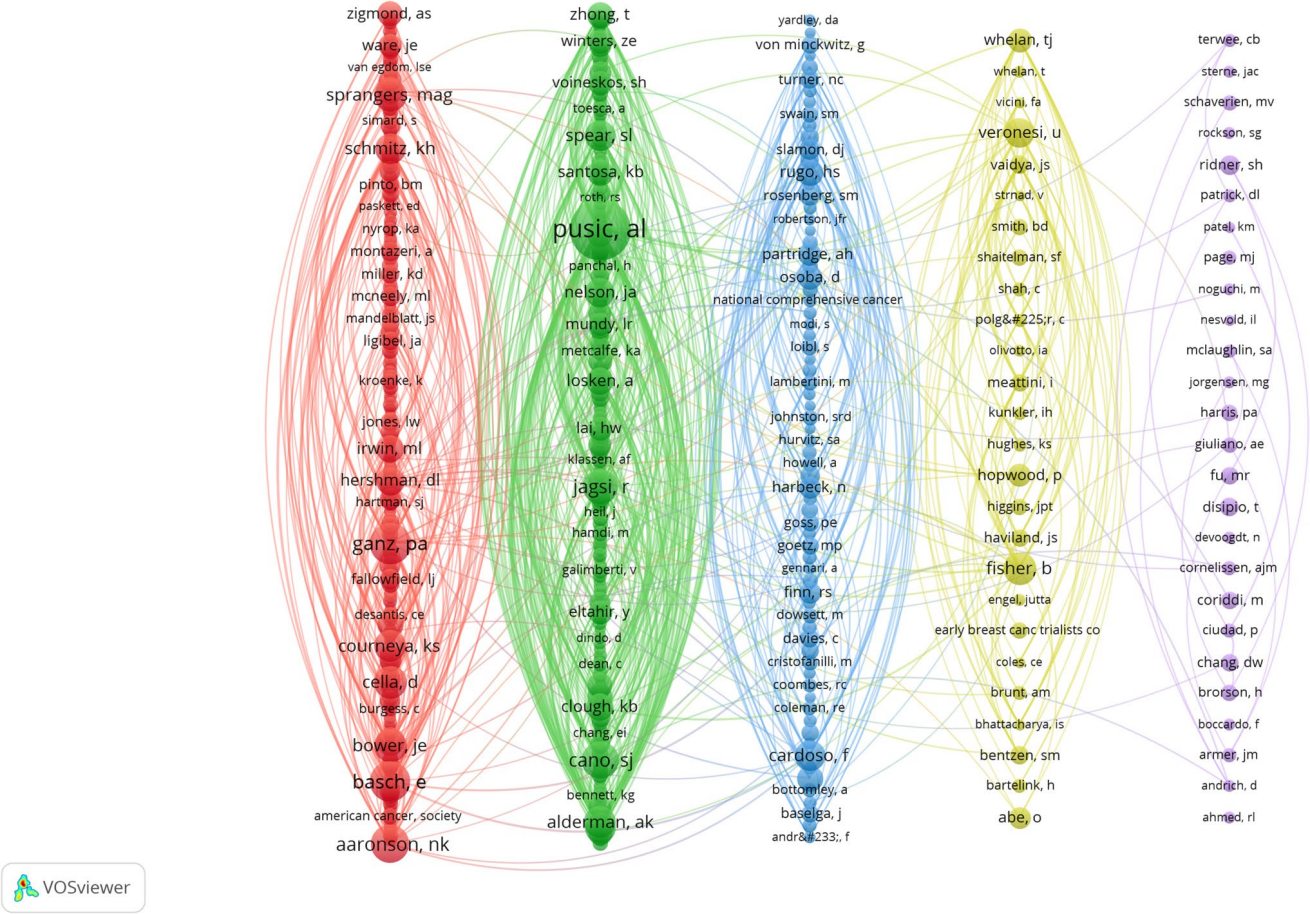
The co-citation network map of authors

Table 5 lists the top 10 authors and co-cited authors by publication volume. While Andrea L. Pusic led in productivity (n = 77) and total citations (5,022), the integration of the average citations per-paper metric reveals a more nuanced landscape of influence. Authors with a smaller publication volume, such as Ji Qi (23 documents) and Hyungjin M. Kim (21 documents), demonstrated exceptional research impact, with the highest average citations of 76.17 and 76.05, respectively. Similarly, Evan Matros (24 documents, 67.46 avg. citations) and Joseph J. Disa (19 documents, 67.47 avg. citations) combined respectable output with high per-article influence. This analysis highlights that total output does not directly equate to the average scholarly impact of an author’s work. CiteSpace visualizes the cooperation network among these authors (Fig. 9). Figure 10 displays the author co-citation network generated by VOSviewer. 254 authors received over 30 citations each. This shows their strong reputation and impact in the field. The largest nodes represent authors who were co-cited with high frequency: A. L. Pusic (n = 545), P. A. Ganz (n = 255), and E. Basch (n = 236).

**Table 5:** Author’s publications and co-citation

Rank	Author	Number of Documents	Total Citations	Average Citations	Rank	Co-cited Author	Citation
1	Pusic, Andrea L	77	5022	65.22	1	Pusic, AL	545
2	Nelson, Jonas A	35	501	14.31	2	Ganz, PA	255
3	Hamill, Jennifer B	29	1891	65.21	3	Basch, E	236
4	Mehrara, Babak J	29	989	34.1	4	Aaronson, NK	212
5	Wilkins, Edwin G	29	1871	64.52	5	Cano, SJ	206
6	Matros, Evan	24	1619	67.46	6	Jagsi, R	179
7	Qi, Ji	23	1752	76.17	7	Bower, JE	161
8	Kim, Hyungjin M	21	1597	76.05	8	Fisher, B	157
9	Morrow, Monica	20	1060	53	9	Cella, D	156
10	Disa, Joseph J	19	1282	67.47	10	Sprangers, Mag	153

### Co-citation references and reference burst

**Fig. 11:**
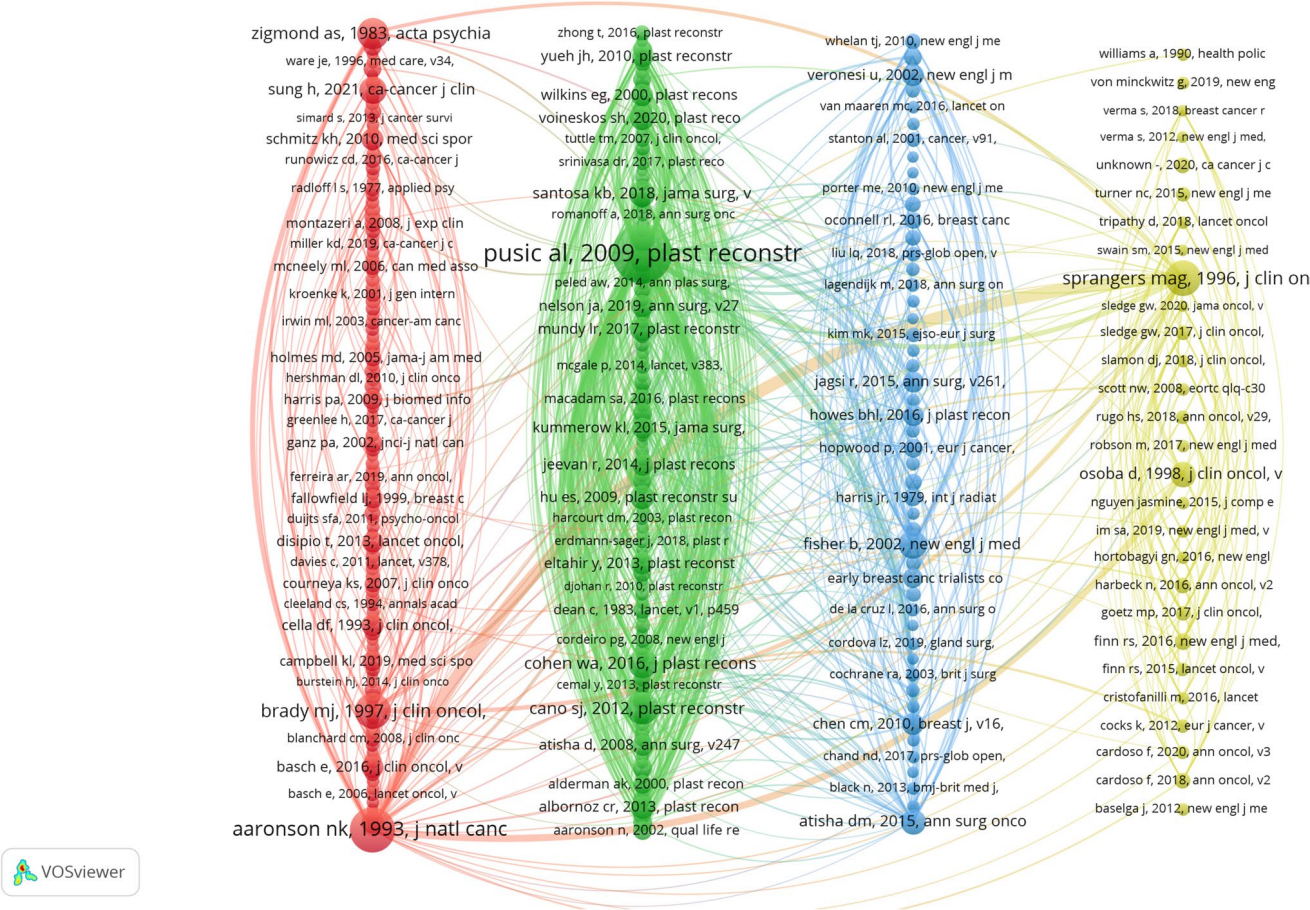
Co-cited network of references

Table 6 and Fig. 11 present the most frequently co-cited articles. The most cited paper, authored by AL Pusic et al. The paper “Development of a New Patient-Reported Outcome Measure for Breast Surgery: The BREAST-Q" [18] was published in Plastic and Reconstructive Surgery in 2009. This study developed the BREAST-Q, a tool for patients to report their satisfaction and quality of life after breast surgery. The tool showed great reliability and validity. This approach filled gaps in current measures by standardizing patient-centered evaluations for clinical decisions and research. The second most co-cited article is “The European Organization for Research and Treatment of Cancer QLQ-C30: A Quality-of-Life Instrument for Use in International Clinical Trials in Oncology" [19]. It was written by N.K. Aaronson et al. and published in the Journal of the National Cancer Institute in 1993. This study aimed to develop a modular tool called the EORTC QLQ-C30. The researchers designed this tool to assess the quality of life for patients with cancer. The third most co-cited article is titled "The European Organization for Research and Treatment of Cancer Breast Cancer-Specific Quality-of-Life Questionnaire Module: First Results From a Three-Country Field Study" by M.A.G. Sprangers [20]. This study created and tested the EORTC QLQ-BR23, a breast cancer-specific quality-of-life questionnaire. It works with the core QLQ-C30 questionnaire.

**Table 6:** The top 10 co-cited references

Rank	Title	Journal	First Author	Total Citations	Year
1	Development of a New Patient-Reported Outcome Measure for Breast Surgery: The BREAST-Q	Plastic and Reconstructive Surgery	Pusic, AL	349	2009
2	The European Organization for Research and Treatment of Cancer QLQ-C30: a quality-of-life instrument for use in international clinical trials in oncology	Journal of the National Cancer Institute	Aaronson, NK	197	1993
3	The European Organization for Research and Treatment of Cancer breast cancer-specific quality-of-life questionnaire module: first results from a three-country field study	Journal of Clinical Oncology	Sprangers, MAG	127	1996
4	Reliability and validity of the Functional Assessment of Cancer Therapy-Breast quality-of-life instrument	Journal of Clinical Oncology	Brady, MJ	123	1997
5	The BREAST-Q Further Validation in Independent Clinical Samples	Plastic and Reconstructive Surgery	Cano, SJ	105	2012
6	The Hospital Anxiety and Depression Scale	Acta Psychiatrica Scandinavica	Zigmond, AS	97	1983
7	The BREAST-Q in surgical research: A review of the literature 2009–2015	Journal of Plastic, Reconstructive & Aesthetic Surgery	Cohen, WA	83	2016
8	Twenty-Year Follow-up of a Randomized Trial Comparing Total Mastectomy, Lumpectomy, and Lumpectomy plus Irradiation for the Treatment of Invasive Breast Cancer	The New England Journal of Medicine	Fisher, B	78	2002
9	Global Cancer Statistics 2020: GLOBOCAN Estimates of Incidence and Mortality Worldwide for 36 Cancers in 185 Countries	CA: A Cancer Journal for Clinicians	Sung, H	73	2021
10	Long-term Patient-Reported Outcomes in Postmastectomy Breast Reconstruction	JAMA Surgery	Santosa, KB	71	2018

**Fig. 12:**
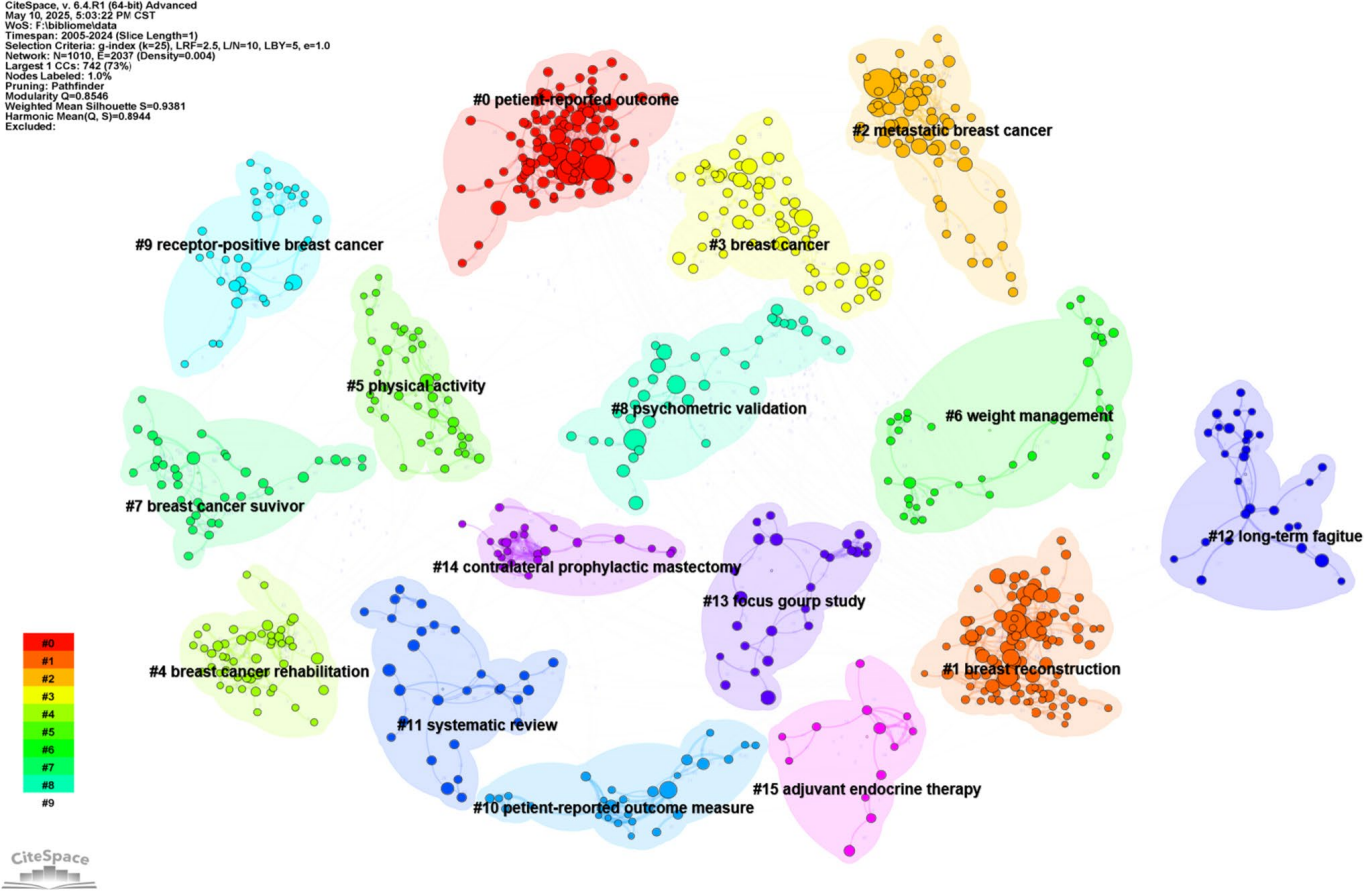
Clustering of co-cited references

**Fig. 13:**
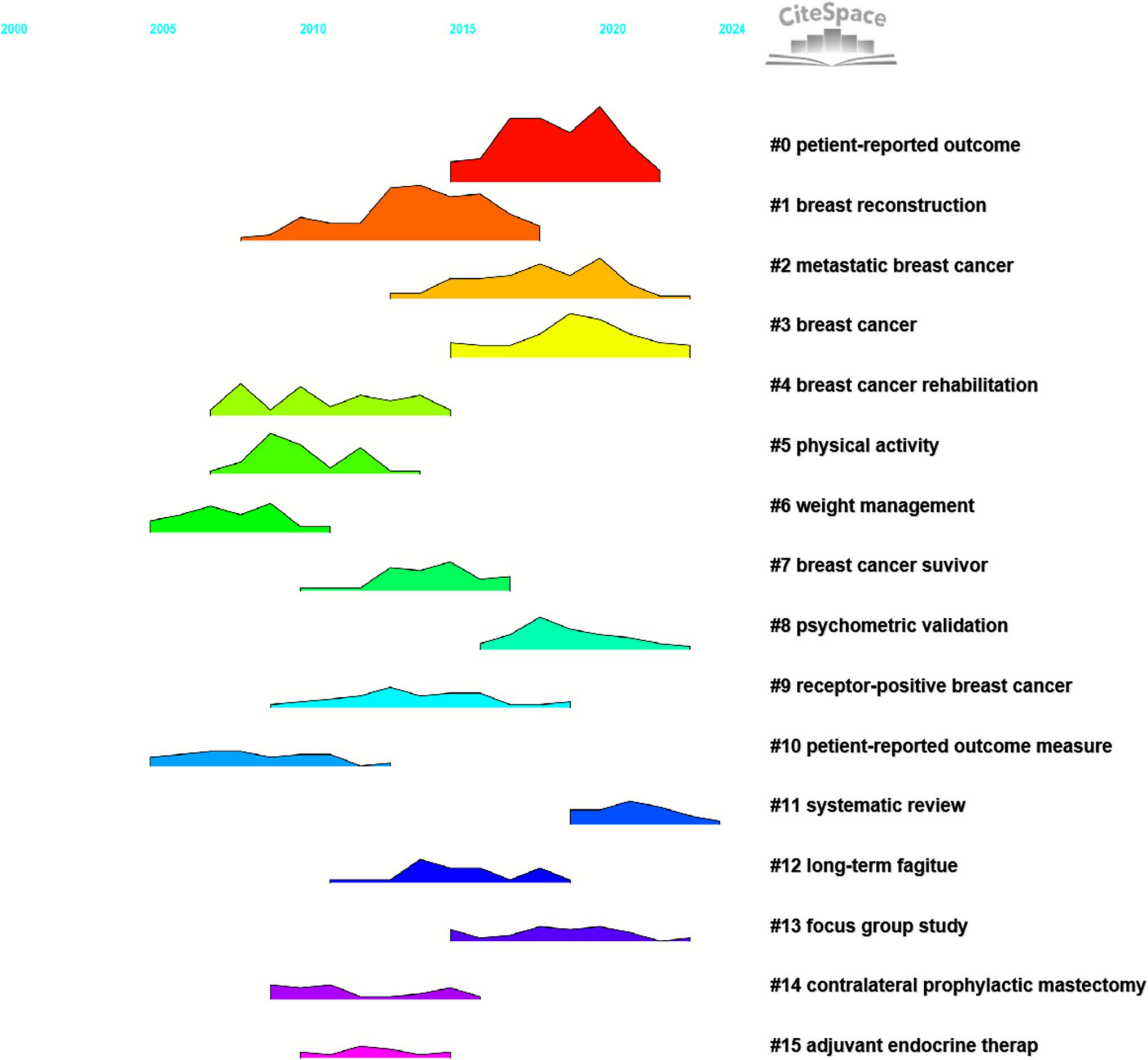
Landscape view of co-cited references

We conducted co-citation reference clustering and temporal clustering analyses, as detailed in Figs. 12 and 13. We found that "patient-reported outcome measure" (cluster 10) and "weight management" (cluster 6) were key topics in early research. Mid-term research mainly focused on breast reconstruction (cluster 1), breast cancer survivor (cluster 7), and hormone receptor-positive breast cancer (cluster 9). Key research trends in recent years include patient-reported outcome (cluster 0), breast cancer (cluster 3), and psychometric validation (cluster 8).

**Fig. 14:**
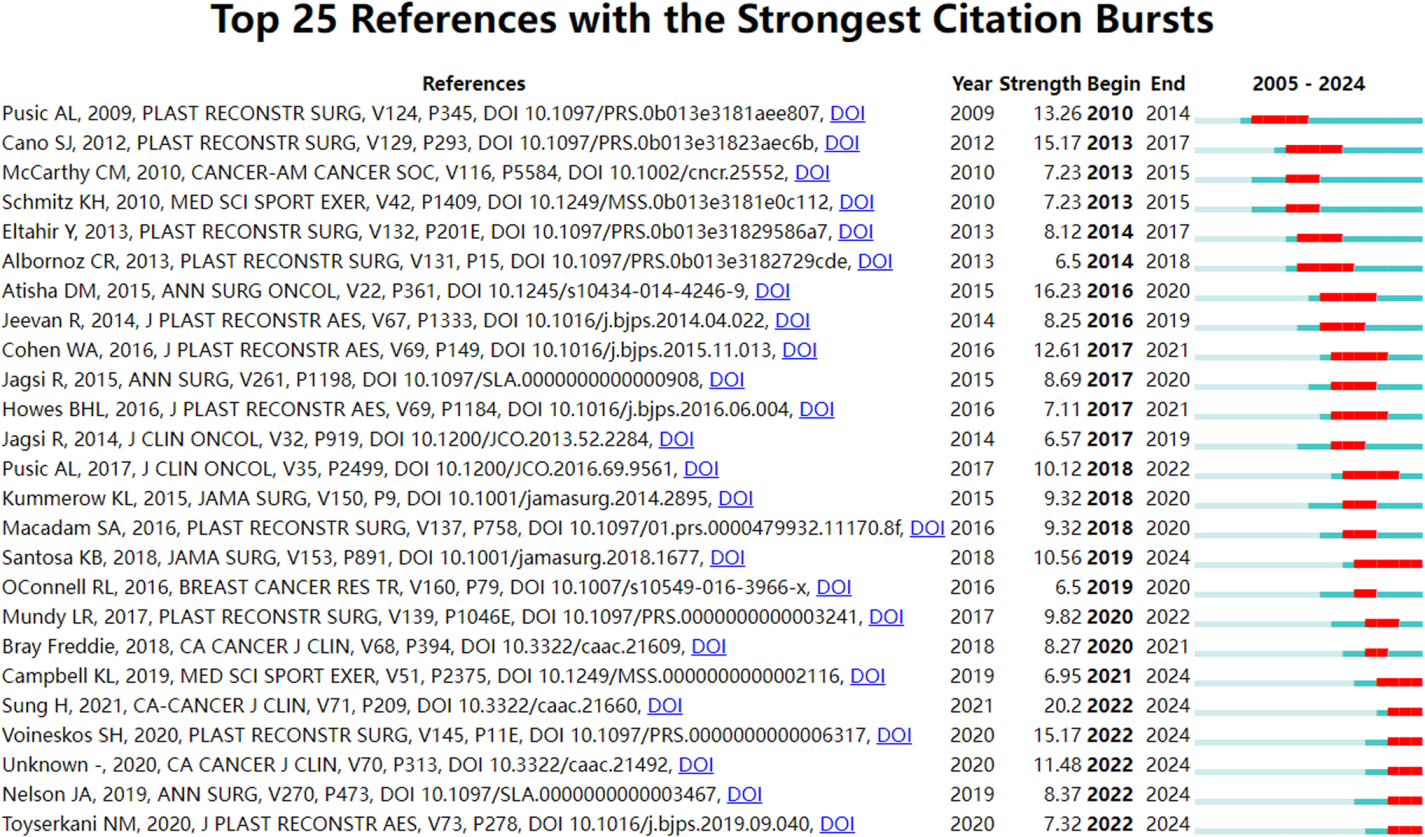
Burst map of cited references

Figure 14 identifies significant citation bursts within breast cancer PROs and QoL literature, marking periods of heightened scholarly influence. The highest burst (20.2) appeared in the article “Global Cancer Statistics 2020: GLOBOCAN Estimates of Incidence and Mortality Worldwide for 36 Cancers in 185 Countries” [21]. This article by H. Sung, published in 2021, is active from 2022 to 2024. It highlights recent progress in survivorship metrics. Earlier studies like D.M. Atisha (2015; 16.23, 2016–2020) and S.J. Cano (2012; 15.17, 2013–2017) [22, 23] focused on surgical outcomes and the validation of PROs. Notable sustained bursts included A.L. Pusic (2009; 13.26, 2010–2014) [18], reflecting an enduring impact. After 2020, bursts like S.H. Voineskos (2020; 15.17) [24] highlighted new themes in reconstruction and psychosocial care. These citation bursts reveal a clear shift toward patient-centered outcomes and innovative methods over the past 20 years.

### Keyword analysis

**Fig. 15:**
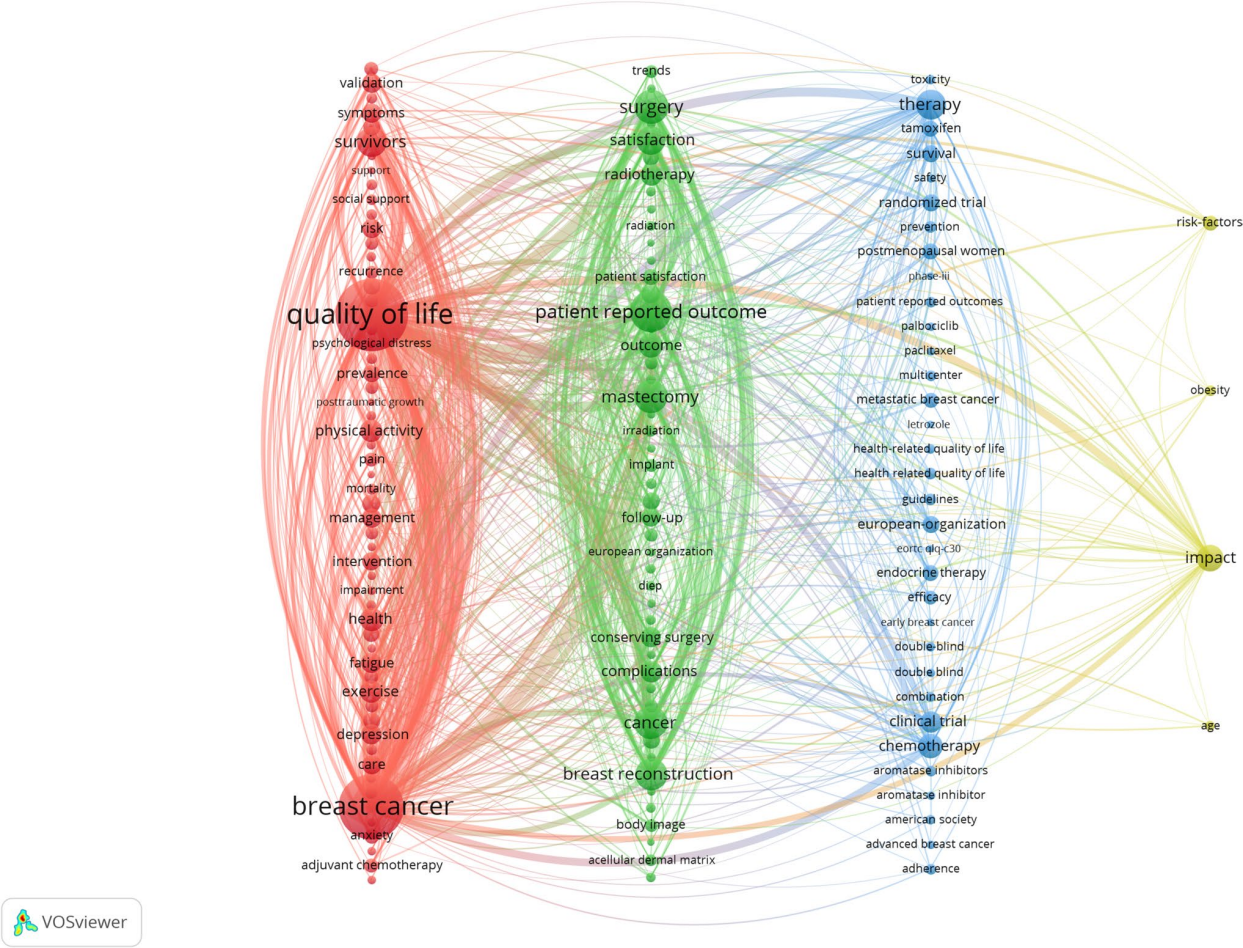
The co-occurrence network map of keywords

**Fig. 16:**
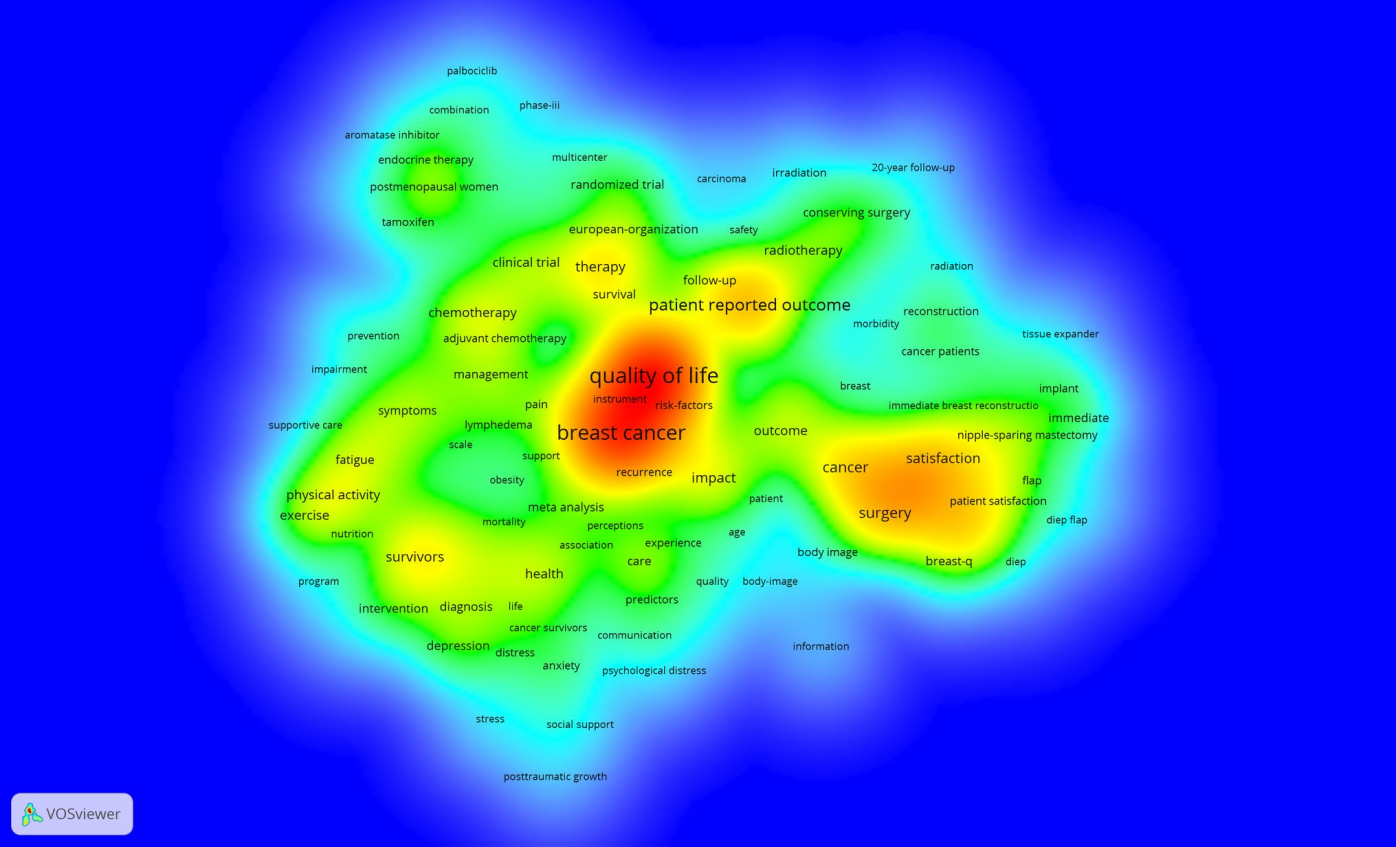
The density map of keywords

Keyword analysis maps research landscapes and trends. After filtering, 142 high-frequency keywords (> 20 occurrences) formed the co-occurrence network. Dominant terms included "quality of life" (n = 1,014), "breast cancer" (n = 811), and "patient-reported outcome" (n = 370) (Table 7), forming four thematic clusters in VOSviewer mapping (Figs. 15 and 16).

**Table 7:** High frequency keyword

Rank	Keyword	Counts	Rank	Keyword	Counts
1	quality of life	1014	11	impact	164
2	breast cancer	811	12	chemotherapy	151
3	patient-reported outcome	370	13	outcome	142
4	surgery	264	14	health	136
5	cancer	255	15	exercise	127
6	mastectomy	254	16	physical activity	119
7	breast reconstruction	242	17	radiotherapy	117
8	survivors	212	18	clinical trial	115
9	therapy	205	19	complications	114
10	satisfaction	199	20	conserving surgery	106

**Fig. 17:**
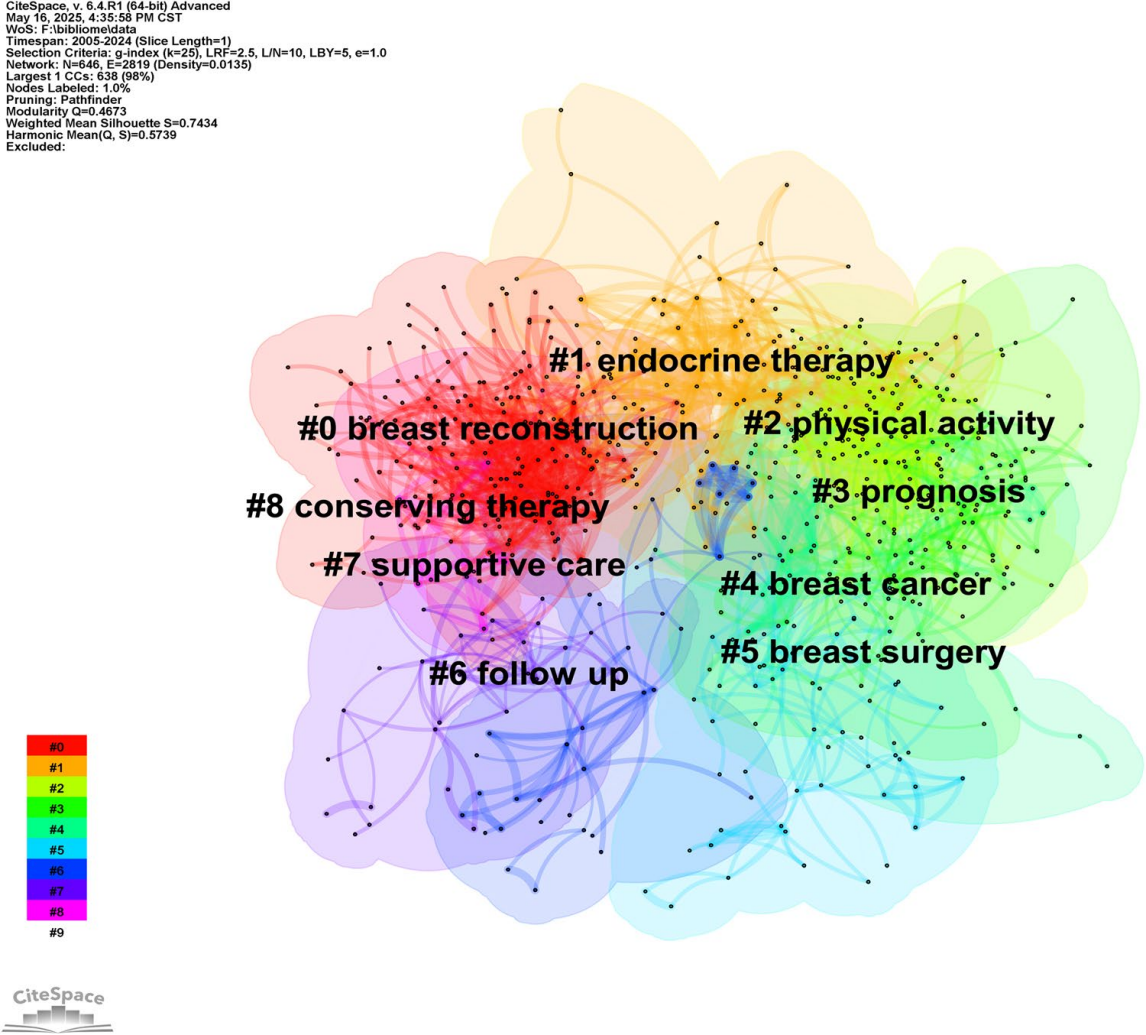
The cluster map of keywords

**Fig. 18:**
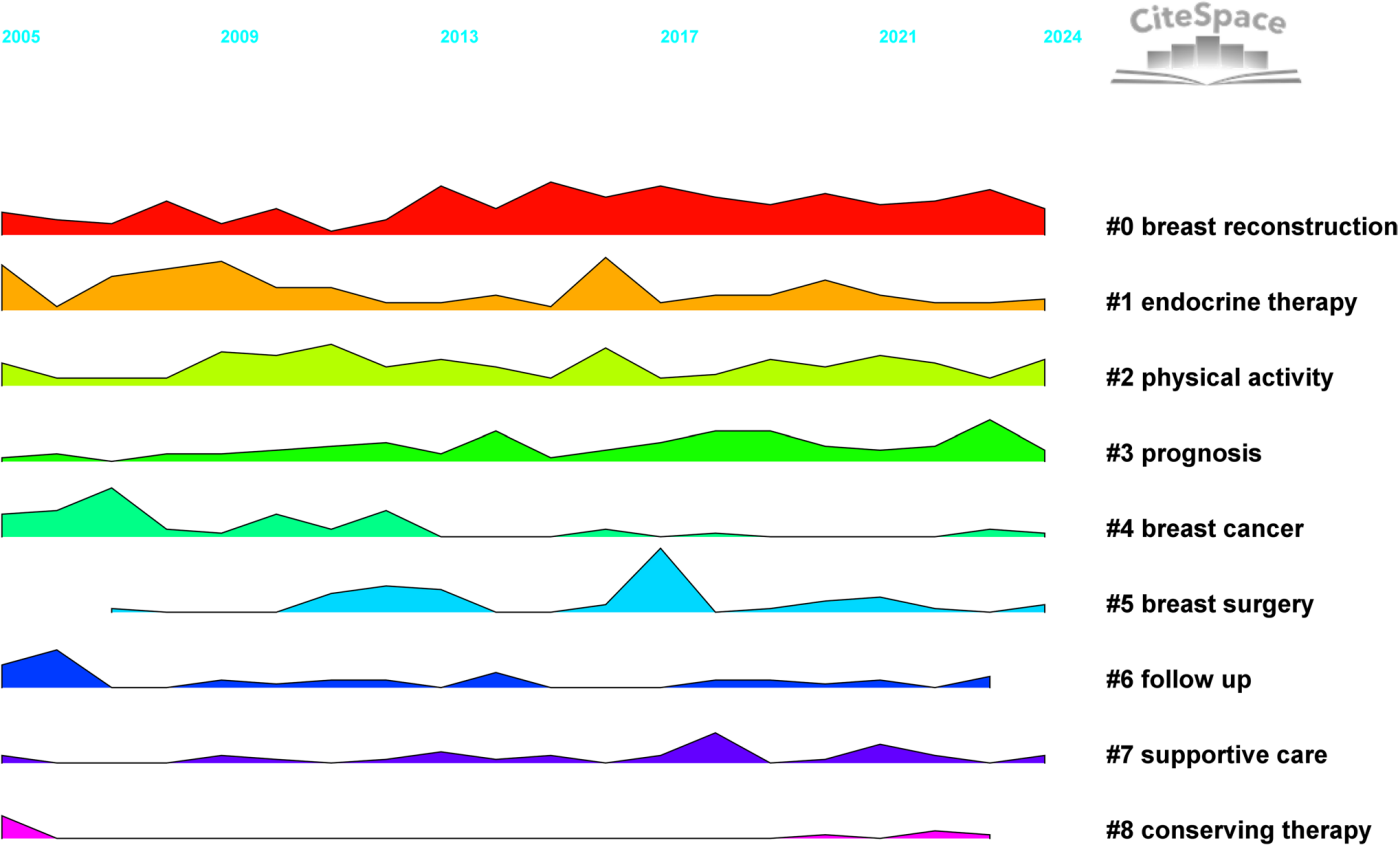
The landscape view of keywords

CiteSpace cluster maps and landscapes (Figs. 17 and 18) visualize evolving research hotspots, highlighting breast reconstruction, endocrine therapy, prognosis, and physical activity as current core research domains.

**Fig. 19:**
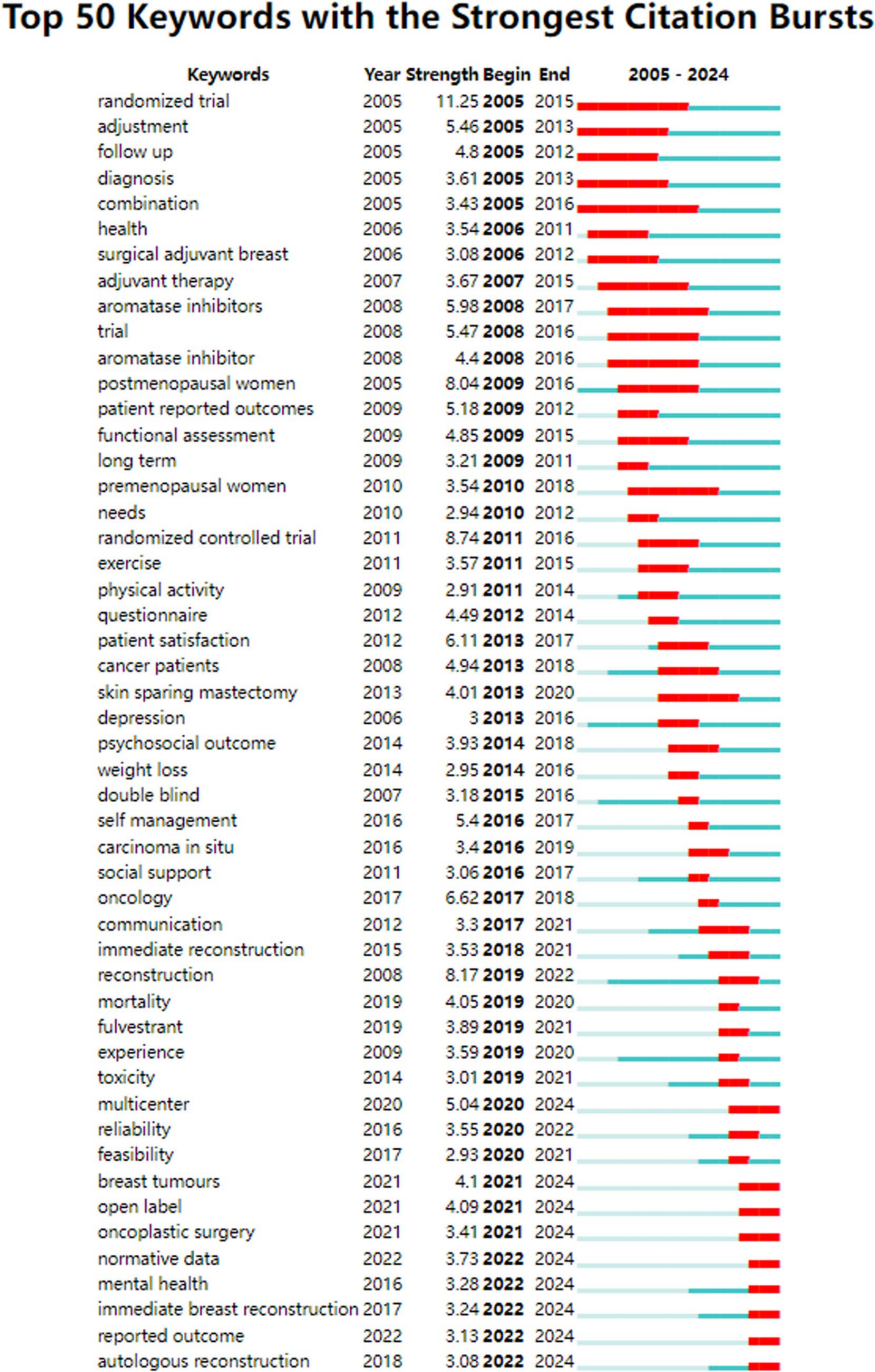
The burst map of keywords

We analyzed the 50 most significant keywords with strong citation bursts (Fig. 19), revealing current research priorities and emerging trends. Burst analysis displayed clear temporal shifts in breast cancer PRO/QoL studies. The early phase (2005–2015) emphasized methodological rigor, featuring "randomized trial" (degree = 46, centrality = 0.11) and "adjuvant therapy" (degree = 35, centrality = 0.05), reflecting a focus on treatment efficacy. In contrast, recent bursts (2020–2024) highlight new directions: "mental health" (degree = 39, centrality = 0.05) and "immediate breast reconstruction" (degree = 13, centrality = 0.01), signaling a shift toward psychosocial well-being and patient-centered outcomes. This evolving landscape balances traditional therapeutic assessment with holistic patient care.

**Fig. 20:**
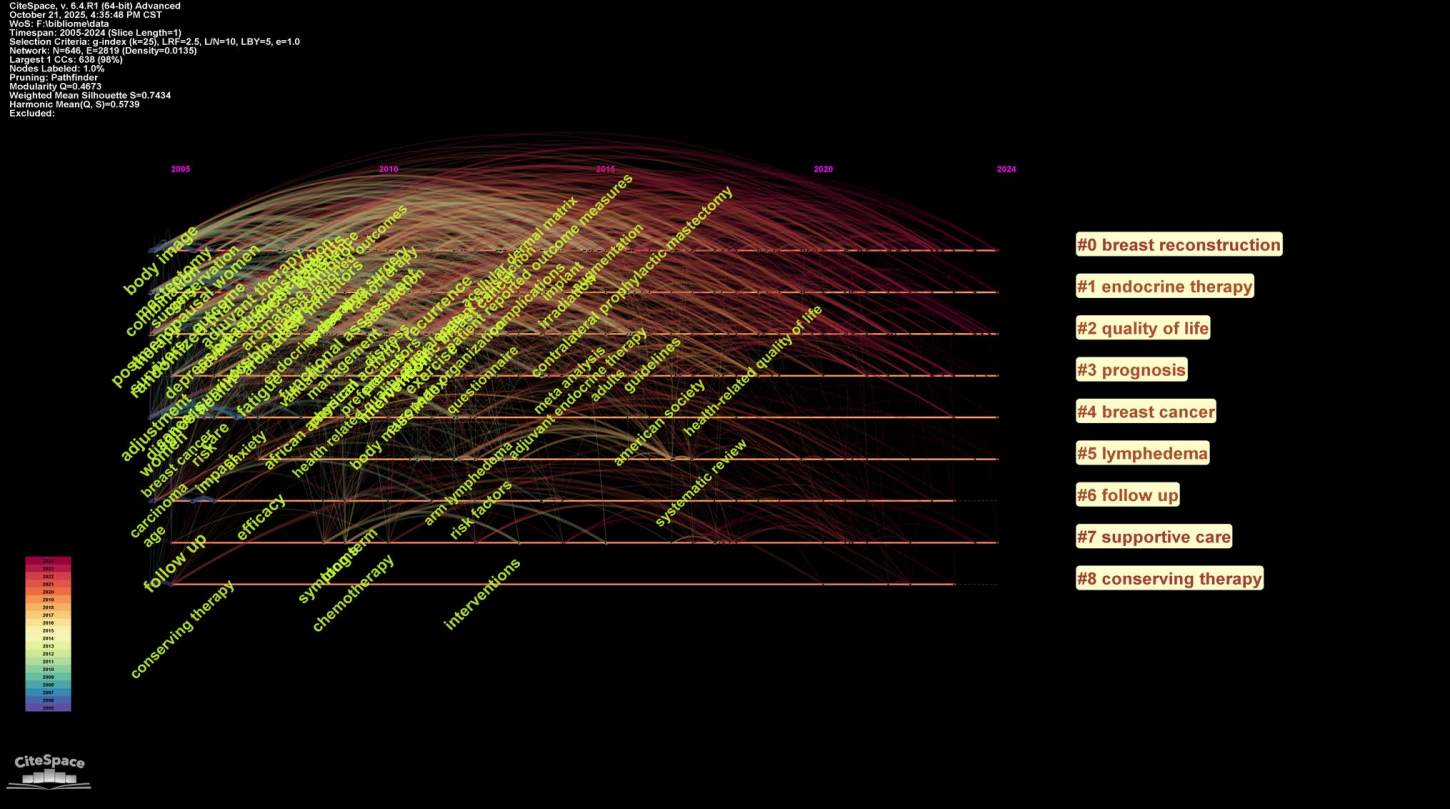
The timeline view of keywords

Timeline and keyword analysis delineated the field’s evolution and its clinical context (Fig. 20). Research progressed from a foundational phase (2005–2012) focused on PRO instrument validation to an intervention-focused phase (2013–2019) dominated by breast reconstruction and survivorship (e.g., exercise), and into the current patient-centric phase (2020–2024) marked by mental health and refined surgical techniques.

The themes distinctly align with primary treatment modalities: PROs in surgical clusters (e.g., Clusters 0, 8) are linked to loco-regional therapies (e.g., mastectomy, reconstruction), while themes in systemic therapy clusters (e.g., Cluster 1) are tied to systemic therapies (e.g., endocrine therapy). The evidence base is predominantly built on Phase 3 RCTs, as indicated by the high burst strength of "randomized trial," supplemented by evidence from large observational cohorts.

## Discussion

The integration of patient-reported outcomes (PROs) and quality-of-life (QoL) assessments in breast cancer research signifies a paradigm shift toward patient-centered oncology care [25]. This trend shows a greater focus on patients’ overall well-being. Over the past two decades, advances in adjuvant therapies and improvements in comprehensive care have substantially improved survival rates for breast cancer. This progress has been accompanied by a growing recognition that patients’ quality of life and subjective experiences, as captured through PROs, are equally critical for delivering comprehensive, personalized care. However, the global growth of PRO-related research has not been systematically tracked. This makes it hard to understand how academic priorities, cross-field collaboration, and regional contributions have shaped the field. Our bibliometric analysis addresses this gap. Our bibliometric analysis addresses this gap. We trace 20-year thematic trends, map knowledge collaboration networks, and identify new research directions. The study reveals the scientific community’s growing focus on two key goals: treatment effectiveness and patient-centered values. Synthesizing these patterns, our study demonstrates PROs’ maturation into evidence-based practice pillars while exposing persistent geographical and topical disparities. These findings necessitate rebalancing research agendas to better address global patient needs.

### Growth in publication output and research interest

Annual publications on PROs and QoL in breast cancer care grew exponentially from 15 (2005) to 274 (2024), cumulating 1,857 studies. Growth accelerated markedly post-2016, doubling output by 2024—reflecting oncology’s patient-centered shift. This expansion was driven by surgical innovations (e.g., oncoplastic techniques) [26], PRO standardization [27], and global survivorship initiatives [28, 29]. Despite pandemic disruptions, publications maintained steady growth (155 in 2020 → 204 in 2021), underscoring sustained commitment to patient-centered outcomes.

However, rapidly increasing publications risk reaching a saturation point if they remain focused on a narrow range of topics. The surge in publications from 2016 to 2024 (from 96 to 274) avoided this risk by diversifying its themes, particularly by refining PRO measures and exploring new research endpoints. Future progress should prioritize methodological optimization and innovative approaches over quantitative expansion. These findings reaffirm the centrality of PROs and highlight that sustained advancement demands high-impact studies with conceptual breakthroughs.

### Countries, institutions, and authors

Our analysis reveals a pronounced dominance of Western nations—particularly the United States and several European countries—in shaping the breast cancer PROs/QoL research landscape, accounting for the majority of global output. This hegemony is underpinned by substantial funding, advanced research infrastructure, and dense international collaboration networks. In stark contrast, Low- and Middle-Income Countries (LMICs), where the burden of breast cancer is rising most rapidly, remain severely underrepresented. This geographical disparity is alarming, as research priorities and findings from high-income countries may not fully address the unique challenges faced in LMIC settings, such as delayed diagnosis, limited treatment access, and distinct survivorship needs [30]. The underrepresentation stems from systemic inequities, including funding disparities, constrained local research capacity, and limited integration into global collaborative networks [31].

Concurrently, our mapping of institutional and author networks indicates significant thematic and collaborative siloing. Research clusters often form around specific domains—such as surgical techniques (e.g., breast reconstruction) or methodological development—with limited cross-cluster interaction [32–35]. While high-productivity institutions and authors in North America and Europe form the core of the network, contributions from other regions, including Asia, and from institutions focusing on psychosocial or survivorship aspects, appear less integrated. This fragmentation potentially limits the cross-fertilization of ideas and the development of truly holistic, patient-centered research that equally values surgical outcomes, psychosocial well-being, and survivorship care.

**Fig. 21:**
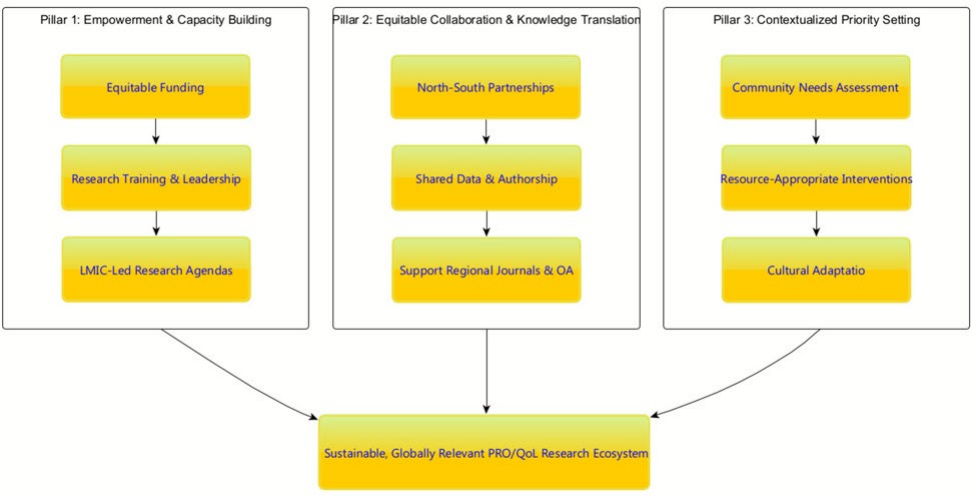
A Conceptual Framework for an Equitable Global PROs/QoL Research Ecosystem

Creating a more equitable and impactful research ecosystem requires shifting leadership and resources to underrepresented regions. To achieve this, we propose a conceptual framework (Fig. 21) with three core components for building a sustainable and globally relevant PROs/QoL research ecosystem. First, Empowerment and Capacity Building requires shifting from isolated project funding to sustained investment in LMIC research infrastructure, including equitable funding streams, research training, and support for locally led research agendas. Second, Equitable Collaboration and Knowledge Translation must replace extractive "helicopter research" with genuine North–South partnerships characterized by shared leadership, data ownership, and authorship, alongside strengthening regional journals and open-access platforms [36–38]. Third, Contextualized Priority Setting ensures that research agendas are driven by community needs assessments, focusing on resource-appropriate interventions and incorporating cultural adaptation. This comprehensive strategy provides a roadmap to transform PRO research from a mirror reflecting global disparities into a tool for advancing global health equity.

The translation of PROs and QoL evidence into routine clinical oncology practice—such as informing shared decision-making, personalizing treatment pathways, and structuring survivorship care plans—remains a critical frontier. While our study maps the scholarly landscape, the direct impact on clinical care pathways warrants greater emphasis in future research and implementation science.

### PROs/QoL trends in breast cancer research

The integration of co-citation networks, keyword co-occurrence analysis, and burst detection offers a multidimensional perspective on the intellectual evolution of PROs and QoL research in breast cancer over the past two decades. Seminal works anchor key co-citation clusters, exemplifying the foundational role of methodological rigor in establishing core surgical techniques and QoL assessment frameworks (e.g., Pusic et al. on breast reconstruction outcomes [18] and Aaronson et al. validating the EORTC QLQ-C30 instrument [19]).

Temporal shifts in research priorities emerge distinctly through co-citation bursts and keyword dynamics. Early research emphasized treatment efficacy and methodological validation (e.g., randomized trials, adjuvant therapy), while recent trends signal a clear shift toward proactive psychosocial-technical innovation and holistic patient care, as seen in emerging bursts related to mental health and immediate reconstruction [21, 39]. This evolution reflects oncology’s broader movement toward integrated, patient-centric models, necessitating methodological advances to capture dynamic PROs effectively throughout the survivorship journey [40].

However, thematic misalignments between established citation networks and emerging keyword trends expose critical research gaps. Co-citation analysis, reflecting historically influential work, heavily emphasizes surgical outcomes and instrument validation. In contrast, keyword analysis reveals growing but still marginal attention to critical psychosocial complexities (e.g., body image, lymphedema) and, most notably, a severe underrepresentation of socioeconomic and equity factors (e.g., cost, racial disparity, care for young women). This indicates a field that excels in technical and methodological validation but lags in addressing the full intersectionality of patient experience, including equity, accessibility, and the specific needs of vulnerable subgroups.

To advance PRO and QoL research, the field must combine its established methodological strengths with a stronger focus on new, patient-centered priorities. Future research must leverage robust citation-driven evidence to validate and implement digital innovations (e.g., eHealth) and design trials that explicitly address intersectional disparities across racial, age, and socioeconomic dimensions. For instance, embedding "racial disparity" into core research frameworks can catalyze culturally tailored interventions, while amplifying focus on "young women" can advance fertility preservation and age-specific survivorship research. Ultimately, aligning the citational authority of methodological foundations with the urgent narratives of patient diversity and equity will transition breast cancer care from a predominantly biomedical model toward an equity-focused holistic paradigm, enhancing lived experiences beyond survival metrics.

### Limitations

This bibliometric study has several limitations. First, by relying solely on the Web of Science database, it may overlook non-English and regional publications from Low- and Middle-Income Countries (LMICs), as well as research with specific cultural contexts. Second, citation-based analysis tends to favor older, established literature (time-lag bias) and reflects academic influence rather than practical clinical impact. Most importantly, this quantitative approach cannot capture unpublished data, detailed patient perspectives, or real-world disparities in Patient-Reported Outcome (PRO) access. Thus, it maps the academic discourse but not the full range of patient experiences or implementation challenges.

Future research should therefore combine bibliometrics with qualitative data from diverse settings, using mixed-methods approaches to improve the translatability and equity focus of the findings.

## Conclusion

This 20-year bibliometric analysis delineates the maturation of PROs and QoL research into a cornerstone of patient-centered breast cancer care, marked by innovations in surgical techniques, validated assessment tools, and survivorship science. However, the field remains constrained by a Western-centric paradigm that perpetuates geographic and thematic inequities. Critical patient needs—including those of racial minorities, young patients concerned with fertility, and populations in resource-limited settings—are systematically underrepresented. Moving forward, the pursuit of truly global and equitable patient-centered care necessitates a fundamental shift toward inclusive research governance that empowers LMIC leadership, contextualized priority-setting that addresses cross-cultural validity gaps, and strategic digital health integration to bridge implementation divides. By embracing these priorities, the future of breast cancer research can transcend survival metrics to meaningfully address the holistic needs of all patients, irrespective of geography or socioeconomic status.

## Supplementary Information

Below is the link to the electronic supplementary material.

Supplementary file1 (DOCX 10 KB)

## Data Availability

No datasets were generated or analyzed during the current study.
