# Supplementary material for: Global trends in patient-reported outcomes and quality-of-life research for breast cancer: a 20-year bibliometric study
Source: Cancer Causes Control. 2026 Jan 17;37(2):20. doi: 10.1007/s10552-025-02097-x (PMC12812104; doi:10.1007/s10552-025-02097-x)
Supplement: Supplementary file 1 — Supplementary file1 (DOCX 10 KB) [file 10552_2025_2097_MOESM1_ESM.docx]

Search Strategy: TS=(("Patient-Reported Outcome*" OR "Patient-Reported Outcome*" OR PROM* OR "Patient Generated Data" OR "Patient-Centered Outcome*") AND ("Quality of Life" OR QoL OR "Health-Related Quality of Life" OR "Life Quality" OR "Well-Being" OR "Satisfaction") AND ("Breast Neoplasm*" OR "Breast Cancer" OR Mastectom* OR "Breast-Conserving Surger*" OR "Breast Reconstruct*" OR "Oncoplastic Surger*" OR "Lymph Node Dissection") NOT ("Lung Cancer" OR "Colorectal Cancer" OR "Prostate Cancer" OR "Ovarian Cancer" OR "Pancreatic Cancer" OR "Melanoma" OR "Leukemia" OR "Lymphoma" OR "Multiple Myeloma" )) NOT TS=("animal model*" OR mice OR rat OR vitro) AND SU=(Medicine OR Surgery OR Oncology).
